# Data-Driven Method to Estimate Nonlinear Chemical Equivalence

**DOI:** 10.1371/journal.pone.0130494

**Published:** 2015-07-09

**Authors:** Michael Mayo, Zachary A. Collier, Corey Winton, Mark A Chappell

**Affiliations:** 1 Environmental Laboratory, US Army Engineer Research and Development Center, Vicksburg, MS, 39183, United States of America; 2 Information Technology Laboratory, US Army Engineer Research and Development Center, Vicksburg, MS, 39183, United States of America; Utah State University, UNITED STATES

## Abstract

There is great need to express the impacts of chemicals found in the environment in terms of effects from alternative chemicals of interest. Methods currently employed in fields such as life-cycle assessment, risk assessment, mixtures toxicology, and pharmacology rely mostly on heuristic arguments to justify the use of linear relationships in the construction of “equivalency factors,” which aim to model these concentration-concentration correlations. However, the use of linear models, even at low concentrations, oversimplifies the nonlinear nature of the concentration-response curve, therefore introducing error into calculations involving these factors. We address this problem by reporting a method to determine a concentration-concentration relationship between two chemicals based on the full extent of experimentally derived concentration-response curves. Although this method can be easily generalized, we develop and illustrate it from the perspective of toxicology, in which we provide equations relating the sigmoid and non-monotone, or “biphasic,” responses typical of the field. The resulting concentration-concentration relationships are manifestly nonlinear for nearly any chemical level, even at the very low concentrations common to environmental measurements. We demonstrate the method using real-world examples of toxicological data which may exhibit sigmoid and biphasic mortality curves. Finally, we use our models to calculate equivalency factors, and show that traditional results are recovered only when the concentration-response curves are “parallel,” which has been noted before, but we make formal here by providing mathematical conditions on the validity of this approach.

## Introduction

Assessing chemical effects spanning the molecular to human scales is a great challenge, and is compounded by the introduction of new chemicals every year. Many of these chemicals and their by-products are emitted into the environment, where they have potential to adversely affect the local wildlife, from reproductive or behavioral impairments to disease and death. Mitigating these burdens requires a systematic and reliable way to quantify relationships between a pollutant’s environmental contamination level and the associated spectrum of potentially adverse biological effects. One such mathematical method is the life cycle assessment (LCA) impact assessment framework [1], which employs an “equivalence model” to express the relative change in an impact metric for a chemical contaminant in terms of a well-studied alternative, termed the reference compound [2, 3], while another application of the equivalency model involves the field of mixtures toxicology [4]. In practice, equivalence models assign a value to a reference chemical concentration, *C*_*ref*_, given an input contaminant concentration, *C*_*con*_, and a number of fixed parameter values; these output values reflect (i) the identity of the reference chemical, and (ii) the details of the mapping between contaminant and reference chemical concentrations.

For some impact metrics, the choice of reference chemical has been standardized. For example, carbon dioxide equivalent units, termed CO_2_-equivalents, are nearly universally used to estimate the “global warming potential” impact metric [5, 6], while phosphate equivalent units, termed PO_4_-equivalents, are commonly used in the assessment of aquatic eutrophication [3] (see Table 1 for examples). For other LCA-based impact categories, such as terrestrial and aquatic toxicology, there is no standardized reference chemical [7]. For example, the IMPACT 2002+ LCA impact assessment (LCIA) method employs triethylene glycol as its reference compound for toxicological effects [8], while the USES-LCA methods express such impacts in terms of 1,4-dichlorobenzene [9].

**Table 1:** Selected Equivalency Factors (Adapted from [2]).

Impact Category	Emission	Equivalency Factor	Reference Chemical
Global Warming	CO_2_	1	CO_2_-equivalents
Global Warming	N_2_O	310	CO_2_-equivalents
Global Warming	CH_4_	21	CO_2_-equivalents
Eutrophication	NO_3_	0.42	PO_4_-equivalents
Eutrophication	N_tot_	0.42	PO_4_-equivalents
Eutrophication	P_tot_	3.06	PO_4_-equivalents
Eutrophication	NH_3_	0.33	PO_4_-equivalents
Eutrophication	NO_x_	0.13	PO_4_-equivalents
Acidification	NH_3_	1.88	SO_2_-equivalents
Acidification	NO_x_	0.7	SO_2_-equivalents
Acidification	SO_2_	1	SO_2_-equivalents
Summer Smog	VOC	0.42	C_2_H_4_-equivalents

While the choice of reference compound determines the parameter values associated with the equivalency model, the actual form of the equations reflects the assumptions used to derive them. Many equivalence models rely on the critical assumption that environmental concentrations are often small enough with respect to total environmental background sources to justify a proportional or linear relationship between concentrations of different chemicals [10]. In an example from LCA, the global warming potential associated with one kilogram of CH_4_ has been suggested as equivalent to 21 kilograms of CO_2_ [11]. However, such relationships oversimplify the complexity and ignore the nonlinearity of the environmental and biological processes affected by exogenous chemicals [12], such as acidification, photochemical smog, ecotoxicity, habitat losses, and biodiversity [13]. Methods exist to linearize manifestly nonlinear dose-response curves to obtain practical equivalency factors for use in standard LCIA methods. One technique involves first selecting a point along the curve, such as the LC_50_ value associated with mortality or survivorship, or an EC_50_ value associated with a sublethal response; next, either a tangent line is drawn to this point (the marginal approach), or a line is more simply established passing through this point and the origin (the “average” approach) [14, 15]. Nevertheless, these approaches obscure the underlying nonlinear processes, which introduce model uncertainty, and represent a serious limitation for the interpretation of results. For example, [16] compared human toxicity characterization factors developed from linear and nonlinear dose-response functions and found that linear functions resulted in estimates of carcinogenic and non-carcinogenic effects that were 21 and 35 times higher, respectively, than effects estimated using nonlinear functions. Moreover, accounting for the nonlinearity in dose-response relationships is especially important when the assumed marginality of emissions compared to background concentrations does not hold, such as for effects at the local scale, or effects arising from acute exposure (e.g., occupational settings) [17]. The capacity of modeling frameworks such as LCA to be effective decision-making aids is therefore diminished [13, 18, 19].

Here we report a data-driven method that exploits the full nonlinearity of concentration-response curves to derive effects-based relationships between concentrations of two chemical compounds. These equivalence relationships are presented in the form of simple equations that require few empirical parameter values. We further provide analytic conditions that define regimes wherein the nonlinear models match with the linear relationships common to traditional equivalency factors. While these equivalence models have been developed and presented from an ecotoxicological angle, the method is general enough that any experimental endpoint which exhibits a concentration-response can be used to establish an equivalency relation between two chemical compounds.

## Methods

Chemical effects often differ according to their molecular structure. This is especially evident in biology, wherein absorption, distribution, metabolism and elimination (ADME) of xenobiotic chemicals is generally chemical and tissue specific [20]. Understanding ADME helps to determine the chain of events linking chemical exposure to effects, which often begins with a molecular initiating event (e.g. receptor-ligand binding), and proceeds through increasing levels of biological organization and scale, terminating with an individual or population level adverse outcome [21, 22]. In toxicology, a common measure of an adverse outcome is mortality, i.e. the ratio, *N*(*t*)/*N*_0_, of the number of organisms that have expired, *N*(*t*), after an exposure time *t*, to the initial population level, *N*_0_. A related measure is survivorship, which is the associated fraction of living organisms within the population, 1- *N*(*t*)/*N*_0_, and may be alternatively reported. These and other experiments provide information for the empirical correlation between a metric, such as mortality/survivorship, and concentration of a chemical exposure, often termed a dose-response function.

**Fig 1:**
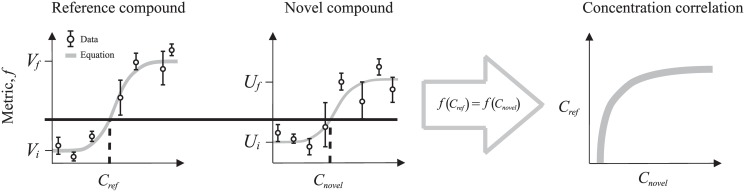
Illustration of the method used to determine chemical equivalence. Concentration-response functions for two chemicals, termed “reference” (left panel) and “novel” (middle panel), can be used to parameterize the relationship between chemical concentrations (right panel).

Such dose-response data can be used to directly derive equations relating concentrations of a “novel” compound to that of a standardized “reference” chemical, based on how they individually affect a common response function (e.g. mortality). Developing these concentration-concentration correlation equations is the primary purpose of this paper. We achieve equations for these correlations by first using a least-squares optimization method to identify the best-fit parameter values associated with an empirical function modeling the dose-response for each chemical. Next, we define an equivalence relation, *f*(*C*_*ref*_) = *f*(*C*_*novel*_), equating values of mortality for each chemical concentration, shown graphically as the solid black horizontal line in Fig 1. Finally, we solve the resulting equation to express the “novel” chemical concentration, *C*_*novel*_, in terms of the “reference” chemical concentration, *C*_*ref*_, and a set of fitted parameter values.

In toxicology, two types of curves model the great majority of mortality-based dose-response curves: monotonic and non-monotonic based functions. In particular, mortality is more often fit to sigmoid-type response functions of the chemical concentration, whereas “biphasic,” or U-shaped responses, are sometimes observed [23]. In specific applications, such as life-cycle impact assessment, a reference chemical may be chosen because the dose-response function behaves reliably, and has been experimentally well-characterized. We therefore assume that a reference chemical has already been identified, and mortality data is available which follows a sigmoid response in the exposure concentration. This assumption reduces the potential number of reference-novel chemical relationships from four to two; thus, in what follows we focus only on determination of the sigmoid-to-sigmoid and biphasic-to-sigmoid concentration-concentration correlation functions.

### Equation for chemical equivalence: sigmoid-sigmoid response functions

An empirical response function commonly used to fit mortality/survivorship data, which is often taken as the response of the reference chemical, is given by a sigmoid equation:

$$\text{Ex:}\;f\left(C_{ref}\right)=V_{i}+\left(V_{f}-V_{i}\right)\frac{{\left(C_{ref}/K_{ref}\right)}^{n}}{1+{\left(C_{ref}/K_{ref}\right)}^{n}}$$ (1)

Here, *V*_*i*_ and *V*_*f*_, are, respectively, the initial and final levels of the experimental response endpoint, such as mortality. In this context, a reference compound may typically be chosen such that empirically *V*_*i*_ = 0 (all living, for *C*_*ref*_ = 0), and *V*_*f*_ = 1 (all deaths, for *C*_*ref*_ = ∞). Finally, *K*_*ref*_ is the concentration that marks the mid-point of the maximum deaths, (*V*_*i*_ + *V*_*f*_)/2 (which may or may not be equal to standard metrics, such as the LC_50_ value; see discussion on normalization below), whereas the parameter *n* quantifies the slope at the sigmoid’s inflection point. In many cases, mortality data for another substance, here termed the “novel” chemical, is also well-fit to a sigmoid equation:

$$\text{Ex:}\;f\left(C_{novel}\right)=U_{i}+\left(U_{f}-U_{i}\right)\frac{{\left(C_{novel}/K_{novel}\right)}^{m}}{1+{\left(C_{novel}/K_{novel}\right)}^{m}}$$ (2)

These parameters have identical meanings to those of Eq 1, but have been labeled to reflect chemical differences in the response function.

As shown in Fig 1, “equivalence” between two different chemical compounds will be understood in terms of their effect on an identical experimental endpoint, such as mortality, which preserves, for example, the species identity and experimental design (e.g. acute or chronic exposures). Such data can then be used to parameterize a relationship between the reference and novel compounds, by simply equating the responses of the concentration-response functions: *f*(*C*_*ref*_) = *f*(*C*_*novel*_). Solving this condition for Eqs 1 and 2 yields an exact concentration-concentration correlation function:

$$\text{Ex:}\;C_{ref}=K_{ref}{\left[\frac{\left(U_{f}-V_{i}\right){\left(C_{novel}/K_{novel}\right)}^{m}+U_{i}-V_{i}}{\left(-U_{f}+V_{f}\right){\left(C_{novel}/K_{novel}\right)}^{m}-U_{i}+V_{f}}\right]}^{1/n}$$ (3)

### Equation for chemical equivalence: sigmoid-biphasic response functions

Non-monotonic dose-response functions, while not as common throughout toxicology as the sigmoid-based response function, have nevertheless been observed for many biological endpoints, such as growth—an effect termed hormesis [24]. While these “biphasic” dose-response functions emerge from a variety of biological mechanisms, they may manifest from molecular-level effects, such as from a competition for active sites between antagonists with differing receptor affinity [25]. At this cellular level, cytotoxicity may emerge to dominate the concentration-response at higher concentrations, an effect that may radically differ from those at the lower concentrations [25]. Such competing influences in the concentration-response, termed biphasic “affectors” [23], may potentially impair signaling events (e.g. phosphorylation/dephosphorylation) through multiple pathways [26], and lead negatively to organism-level reproductive impairments or death, and ultimately, to population decline.

Beckon et al. [23] assumed that such positive and negative affectors contributed separately to the overall concentration-response curve, but relied on the value of a threshold concentration for affector “sensitivity.” If these positive and negative effects coordinate independently, and if the sensitivity thresholds can be taken as sigmoid-type equations of the dose/exposure concentration—as justified in [23] by heuristic arguments, then Beckon *et al*. argued that a concentration-response for the novel chemical may be expressed by the equation:

$$\text{Ex:}\;f\left(C_{novel}\right)=\frac{\left[1+{\left(C_{novel}/K_{novel}^{-}\right)}^{m^{-}}\right]\left[U_{\text{max}}+U_{f}{\left(C_{novel}/K_{novel}^{+}\right)}^{m^{+}}\right]-U_{\text{max}}+U_{i}}{\left[1+{\left(C_{novel}/K_{novel}^{-}\right)}^{m-}\right]\left[1+{\left(C_{novel}/K_{novel}^{+}\right)}^{m^{+}}\right]}$$ (4)

**Fig 2:**
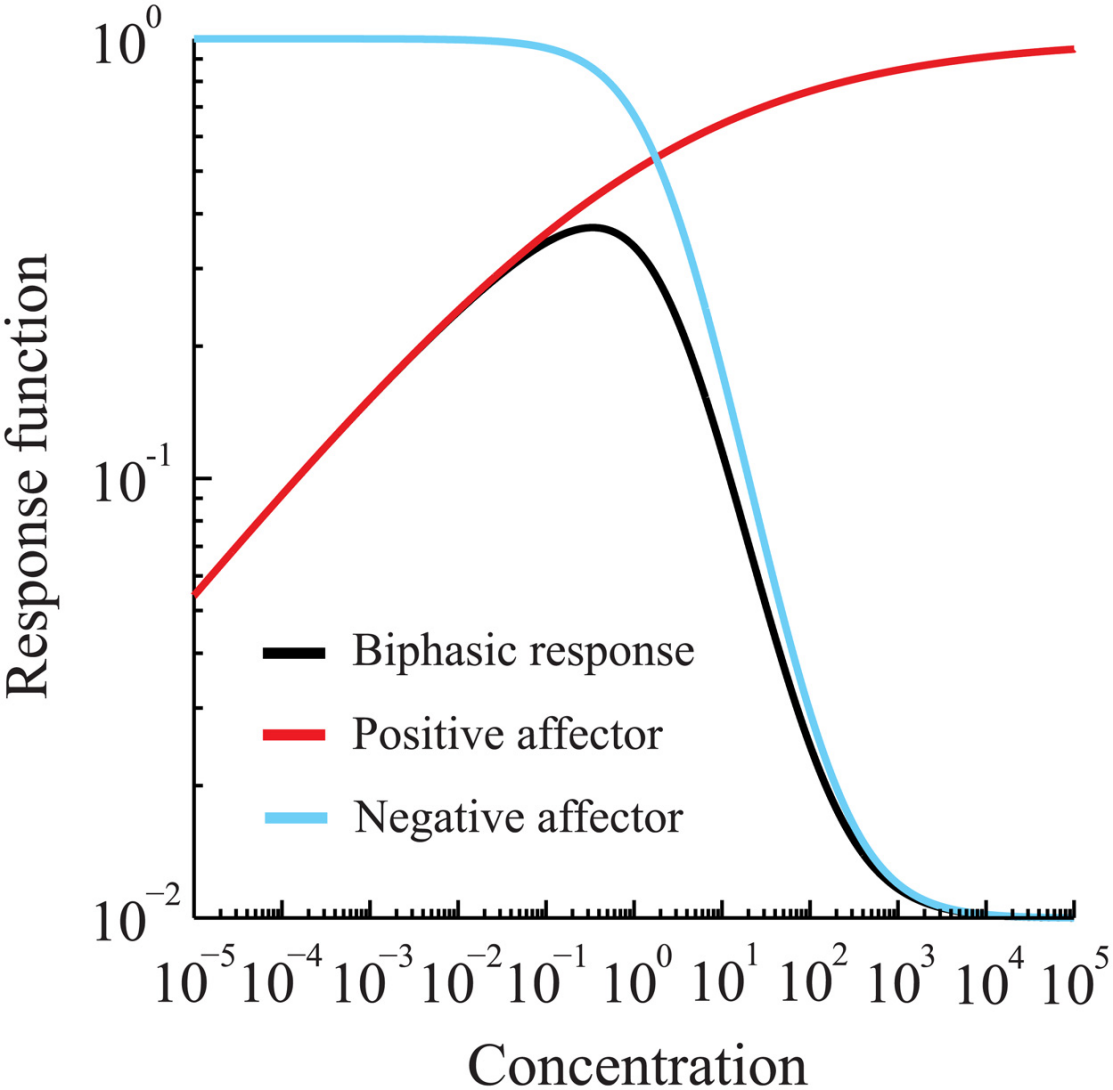
Non-monotone, or “biphasic,” response function. Positive (red line) and negative (blue line) affectors combine to result in a biphasic response function.

We have written this equation in terms of approximate “lower” (-) and “upper” (+) sigmoid-like components of the biphasic response, illustrated in Fig 2, which can be delineated by a concentration: $C_{novel}^{-/+}=\sqrt{K_{novel}^{-}K_{novel}^{+}}$(Eq 7 below, and S1 File). Conceptually this may correspond to regimes wherein, e.g., a toxic response differs mechanistically between lower concentrations ($C_{novel}\leq C_{novel}^{-/+}$) and higher concentrations ($C_{novel}>C_{novel}^{-/+}$). If these mechanisms exist and are mostly independent of one another, then each “half” of the biphasic relationship can be modeled approximately as a sigmoid response, and is representative of cumulative exposure effects.

In Eq 4, the parameters $K_{novel}^{-}$ and $K_{novel}^{+}$ label, respectively, the mid-point concentrations of the lower and upper sigmoid-based affector components (e.g., a median-effect concentration, EC_50_, for each “positive” and “negative” cumulative response); the parameters $m^{-}$ and $m^{+}$, respectively, help to quantify the slope at the mid-point concentration, in the sense that larger values represent a more switch-like response; *U*_*i*_ and *U*_*f*_ are the initial and final levels of the biphasic dose-response curve; and *U*_max_ is the theoretical maximum (minimum) reached by the positive (negative) affector component free of any contribution from the competing affector.

Now, if we naively follow the procedure of the previous section that led to Eq 3 (Fig 1)—first, by defining an equivalence relation for the fully nonlinear concentration-response function, and then using Eq 4 in place of Eq 2—then it would quickly become apparent that two novel chemical concentrations could potentially be associated with a singular concentration of the reference chemical. To solve this problem, we decomposed the fitted biphasic equation for the novel chemical concentration-response, Eq 4, into constituent parts: a lower, *f* ^*−*^(*C*_*novel*_), and upper, *f* ^*+*^(*C*_*novel*_), component, which can be separated by a threshold concentration mentioned above (Eq 7),$C_{novel}^{-/+}$: *f*(*C*_*novel*_) = *f* ^*−*^(*C*_*novel*_) for $C_{novel}\leq C_{novel}^{-/+}$, and *f*(*C*_*novel*_) = *f* ^*+*^(*C*_*novel*_) for $C_{novel}>C_{novel}^{-/+}$. (We note that our labeling scheme for the decomposition of Eq 4 ignores whether the lower or upper components have been influenced by either the positive or negative affectors).

We now claim that both the lower and upper components may be approximated by sigmoid equations similar to Eq 2. Solving the relevant equivalence equations yields a piecewise solution to the full concentration-concentration relationship (Appendix in A S1 File):

$$\text{Ex:}\;C_{ref}=K_{ref}{\left[\frac{\left({\tilde{U}}_{\text{max}}^{eff,-}-V_{i}\right){\left(C_{novel}/{\tilde{K}}_{novel}^{-}\right)}^{{\tilde{m}}^{-}}+U_{i}-V_{i}}{\left(-{\tilde{U}}_{\text{max}}^{eff,-}+V_{f}\right){\left(C_{novel}/{\tilde{K}}_{novel}^{-}\right)}^{{\tilde{m}}^{-}}-U_{i}+V_{f}}\right]}^{1/n},\text{for }C_{novel}\leq C_{novel}^{-/+},$$ (5)

and

$$\text{Ex:}\;C_{ref}=K_{ref}{\left[\frac{\left(U_{f}-V_{i}\right){\left(C_{novel}/{\tilde{K}}_{novel}^{+}\right)}^{\tilde{m}+}+{\tilde{U}}_{\text{max}}^{eff,+}-V_{i}}{\left(-U_{f}+V_{f}\right){\left(C_{novel}/{\tilde{K}}_{novel}^{+}\right)}^{\tilde{m}+}-{\tilde{U}}_{\text{max}}^{eff,+}+V_{f}}\right]}^{1/n},\text{for }C_{novel}\leq C_{novel}^{-/+}.$$ (6)

In view of this sigmoid interpretation of the full biphasic response function, the threshold concentration, $C_{novel}^{-/+}$, can be approximated by the geometric mean of the mid-point parameters $K_{novel}^{-}$ and $K_{novel}^{+}$ (refer to Appendix A in S1 File):

$$\text{Ex:}\;C_{novel}^{-/+}=\sqrt{K_{novel}^{-}K_{novel}^{+}}$$ (7)

Also shown in the Supporting Information (S1 File), several parameters of Eqs 5 and 6 can be written entirely in terms of the fitted parameters identified for the biphasic response function, Eq 4. These effective exponents of the sigmoid models, ${\tilde{m}}^{-}$ and ${\tilde{m}}^{+}$, can be expressed with the equations:

$$\text{Ex:}\;{\tilde{m}}^{-}=m^{-}\times \frac{\sqrt{{\tilde{U}}_{\text{max}}^{eff,-}/U_{i}}+1}{\sqrt{{\tilde{U}}_{\text{max}}^{eff,-}/U_{i}}-1}\times \frac{\sqrt{U_{\text{max}}/U_{i}}-1}{\sqrt{U_{\text{max}}/U_{i}}+1},\text{ and}$$ (8)

$$\text{Ex:}\;{\tilde{m}}^{+}=m^{+}\times \frac{\sqrt{{\tilde{U}}_{\text{max}}^{eff,+}/U_{f}}+1}{\sqrt{{\tilde{U}}_{\text{max}}^{eff,+}/U_{f}}-1}\times \frac{\sqrt{U_{\text{max}}/U_{f}}-1}{\sqrt{U_{\text{max}}/U_{f}}+1}.$$ (9)

We have assumed that *U*_*f*_, *U*_*i*_ > 0; how to handle nonzero values is explained in the Supporting Information (Appendix B in S1 File). Here, the parameters ${\tilde{U}}_{\text{max}}^{eff,-}$ and ${\tilde{U}}_{\text{max}}^{eff,+}$ express a saturating value ($C_{novel}^{-/+}=\infty$) of the model sigmoid equations, respectively modeling the left- and right-hand sides of the biphasic response. The other effective parameters are:

$$\text{Ex:}\;{\tilde{U}}_{\text{max}}^{eff,-}={\tilde{U}}_{\text{max}}{\left(1+\chi^{-}\sqrt{\frac{{\tilde{U}}_{\text{max}}}{U_{i}}}\right)}^{1/\mu^{-}},\text{ with}$$ (10)

$$\text{Ex:}\;\chi^{-}={\left[{\sqrt{\frac{K^{-}}{K^{+}}}}^{m^{-}}\left(\frac{\sqrt{{\tilde{U}}_{\text{max}}/U_{i}}-1}{U_{\text{max}}/U_{i}-\sqrt{{\tilde{U}}_{\text{max}}/U_{i}}}\right)\right]}^{\frac{\sqrt{U_{\text{max}}/U_{i}}-1}{\sqrt{U_{\text{max}}/U_{i}}+1}\times \frac{\sqrt{{\tilde{U}}_{\text{max}}/U_{i}}+1}{\sqrt{{\tilde{U}}_{\text{max}}/U_{i}}-1}},\text{ and}$$ (11)

$$\text{Ex:}\;\mu^{-}=\frac{{\tilde{U}}_{\text{max}}/U_{i}}{{\tilde{U}}_{\text{max}}/U_{i}-1}\times \frac{\chi^{-}\left(\text{ln}\chi^{-}-1\right)+1}{1+\chi^{-}\sqrt{{\tilde{U}}_{\text{max}}/U_{i}}}.$$ (12)

A similar method can be used to find ${\tilde{U}}_{\text{max}}^{eff,+}$ (for $C_{novel}>C_{novel}^{-/+}$):

$$\text{Ex:}\;{\tilde{U}}_{\text{max}}^{eff,+}=U_{f}{\left(\frac{\chi^{+}\sqrt{U_{f}/{\tilde{U}}_{\text{max}}}+1}{\chi^{+}\sqrt{{\tilde{U}}_{\text{max}}/U_{f}}+1}\right)}^{1/\mu^{+}}{\left(\frac{{\tilde{U}}_{\text{max}}}{U_{f}}\right)}^{\left(\mu^{+}+1\right)/\mu^{+}},\text{ with}$$ (13)

$$\text{Ex:}\;\chi^{+}={\left[{\sqrt{\frac{K^{+}}{K^{-}}}}^{m^{-}}\left(\frac{U_{\text{max}}/U_{f}-\sqrt{{\tilde{U}}_{\text{max}}/U_{f}}}{\sqrt{{\tilde{U}}_{\text{max}}/U_{f}}-1}\right)\right]}^{\frac{\sqrt{{\tilde{U}}_{\text{max}}/U_{f}}+1}{\sqrt{{\tilde{U}}_{\text{max}}/U_{f}}-1}\times \frac{\sqrt{U_{\text{max}}/U_{f}}-1}{\sqrt{U_{\text{max}}/U_{f}}+1}},\text{ and}$$ (14)

$$\text{Ex:}\;\mu^{+}=\frac{\chi^{+}\sqrt{{\tilde{U}}_{\text{max}}/U_{f}}}{\chi^{+}\sqrt{{\tilde{U}}_{\text{max}}/U_{f}}+1}\left(1-\frac{\text{ln}\chi^{+}+1+\sqrt{{\tilde{U}}_{\text{max}}/U_{f}}}{\chi^{+}+\sqrt{{\tilde{U}}_{\text{max}}/U_{f}}}\right).$$ (15)

A value for the local extremum (maximum/minimum) of the biphasic curve can be found by evaluating Eq 4 at the threshold concentration of Eq 7:

$$\text{Ex:}\;{\tilde{U}}_{\text{max}}=\frac{\left(1+{\sqrt{K^{+}/K^{-}}}^{m^{-}}\right)\left(U_{\text{max}}+U_{f}{\sqrt{K^{-}/K^{+}}}^{m^{+}}\right)-U_{\text{max}}+U_{i}}{\left(1+{\sqrt{K^{+}/K^{-}}}^{m^{-}}\right)\left(1+{\sqrt{K^{-}/K^{+}}}^{m^{+}}\right)}.$$ (16)

Finally, new mid-point parameters of the sigmoid equations modeling each side of the biphasic response can be given by:

$$\text{Ex:}\;{\tilde{K}}^{-}=K^{-}{\left(\frac{\sqrt{{\tilde{U}}_{\text{max}}/U_{i}}-1}{U_{\text{max}}/U_{i}-\sqrt{{\tilde{U}}_{\text{max}}/U_{i}}}\right)}^{1/m^{-}}{\left(\frac{{\tilde{U}}_{\text{max}}^{eff,-}/U_{i}-\sqrt{{\tilde{U}}_{\text{max}}/U_{i}}}{\sqrt{{\tilde{U}}_{\text{max}}/U_{i}}-1}\right)}^{1/{\tilde{m}}^{-}},\text{ and}$$ (17)

$$\text{Ex:}\;{\tilde{K}}^{+}=K^{+}{\left(\frac{U_{\text{max}}/U_{f}-\sqrt{{\tilde{U}}_{\text{max}}/U_{f}}}{\sqrt{{\tilde{U}}_{\text{max}}/U_{f}}-1}\right)}^{1/m^{+}}{\left(\frac{\sqrt{{\tilde{U}}_{\text{max}}/U_{f}}-1}{{\tilde{U}}_{\text{max}}^{eff,+}/U_{f}-\sqrt{{\tilde{U}}_{\text{max}}/U_{f}}}\right)}^{1/{\tilde{m}}^{+}}.$$ (18)

We note that these analytic Eqs (5–18) provide a close approximation to the full biphasic response (absolute relative error <5.6%, see Fig B in S1 File), but are written entirely in terms of the fitted parameters of the original dose-response function. Thus, no “re-fitting” of any experimental data is necessary beyond that of the original biphasic curve-fit (Eq 4).

### Examples from the experimental literature

We applied the above methods to several experimental datasets, wherein we derived concentration-concentration functions from the toxicological survivorship data using the sigmoid-to-sigmoid and biphasic-to-sigmoid approaches. Where possible we have employed data from a single literature source to ensure its reproducibility, wherein the chemicals were exposed to the same or similar species under similar time periods and experimental conditions.

#### Sigmoid-to-sigmoid relationship

Survivorship measurements were reported from experiments carried out by LeBlanc and Surprenant [27], wherein they compared the toxicity of three chemicals: dimethyl formamide, acetone, and triethylene glycol (the reference chemical). Several populations of the water flea (*Daphnia magna*) were exposed to constant concentrations of these individual chemicals for 28 days, and the survivors were counted at days 7, 14, 21, and 28 days. The domains of exposure concentrations were similar for all three chemicals, spanning from 600 to 22,000 μl/L, and additionally included a control group.

**Fig 3:**
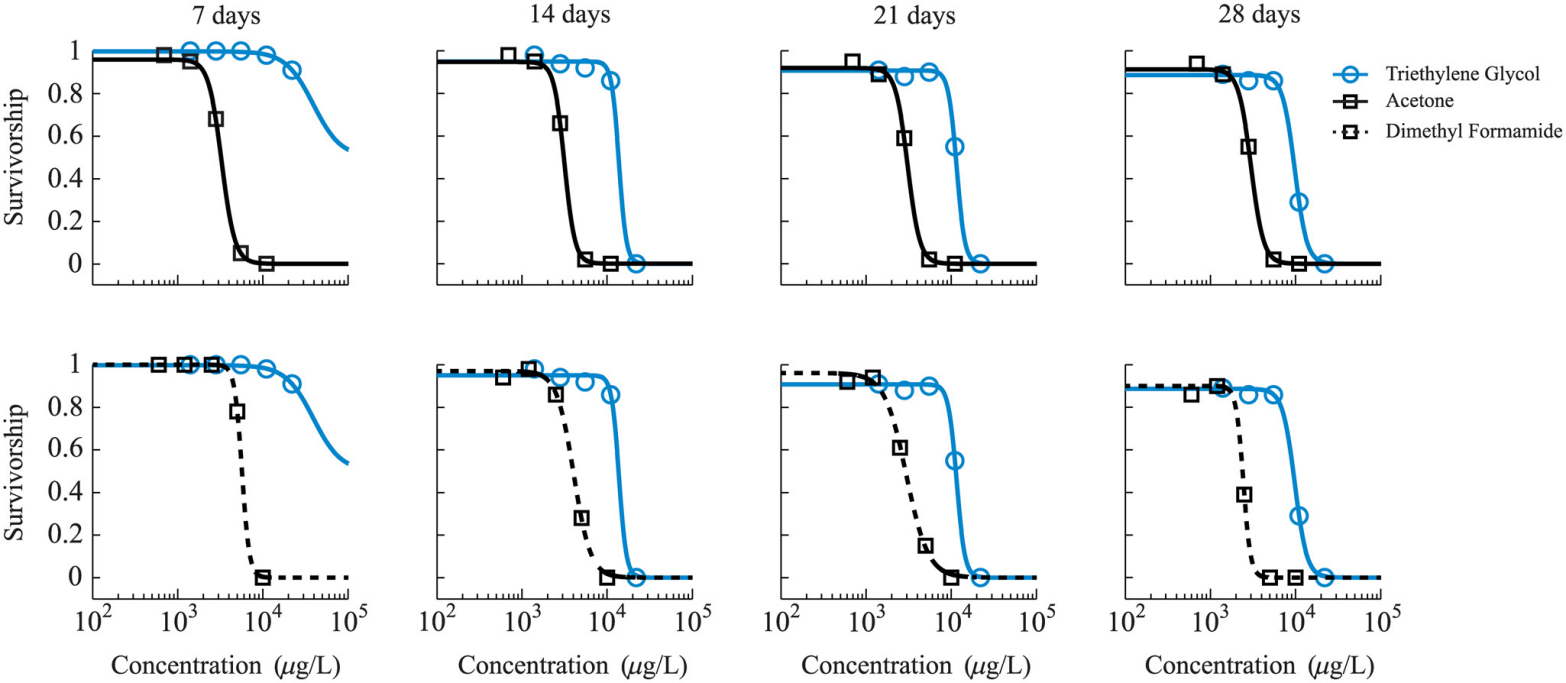
Survivorship data for *Daphnia magna*. Experimental concentration-response data from [25] carried out on the water flea *Daphnia magna*, illustrating sigmoid survivorship curves. These data were fit to empirical sigmoid equations (solid and dotted lines).

As shown by Fig 3, these survivorship data were numerically fit to equations of type (2) by minimizing the least squares residual *F*(*U*_*i*_, *U*_*f*_, *K*, *m*), calculated from the difference between the experimental data and the sigmoid curve:

$$\text{Ex:}\;F\left(U_{i},U_{f},K,m\right)=\sum_{j}{\left[d_{j}-f\left(C_{j},U_{i},U_{f},K,m\right)\right]}^{2}.$$ (19)

Here *U*_*i*_, *U*_*f*_, *K*, and *m* are the parameters of Eq 2, *d*_*j*_ are the response data of the dose-response at chemical concentration *C*_*j*_, and *f* is the output of the empirical sigmoid curve evaluated at the (exposure) concentration value *C*_*j*_.

We used a bounded Nelder-Mead Simplex search method [28] to identify parameter values which produce a sigmoid curve best-fit to the experimental data. The Nelder-Mead algorithm employs a grid-based search algorithm, in contrast to traditional gradient-based methods. One advantage of this method is that the Simplex technique can be more efficiently implemented, as it avoids a need for calculating accurate gradients. However, a search based on the Simplex method often requires significantly more function calls, which can slow computing time. For our purposes the Nelder-Mead algorithm was not computationally prohibitive, wherein the algorithm identified best-fit parameter values in time-scales on the order of tenths of seconds. Specifically, the average compute-time for a solution was approximately 0.07 sec, with a max of 0.18 seconds and a min of 0.03 seconds.

The variable most sensitive to bounding was the Hill-type exponent *m*, as some data points were nearly step functions. In such cases, *m* would trend as large as possible to produce as steep a drop-off from full survival to full mortality. We bounded the exponent to a maximum value of 10 as that still provided a suitable step-function behavior.

In all cases, our initial iterates for the variables were based on common insight to the problem. The parameter *U*_*i*_ was bounded on the (closed) interval [75, 100] with an initial iterate of 90, because survival rates for the control group were always observed to be above 90%. Additionally, the parameter *U*_*f*_ was bounded on the interval [0,25], with an initial iterate of 10, chosen to reflect our observation from the data that populations nearly always achieved full mortality at the highest exposure concentrations. The Hill-type exponent of the sigmoid model was, as discussed previously, bounded on the interval [0,10]; we chose an initial iterate of 1 to reflect a smoother transition from full survival to full mortality. Finally, a value for the parameter *K* was bounded to [0,2 × max(*C*)], wherein max(*C*) represents the highest exposure concentration that was observed from the given experimental dataset. We fixed the initial iterate to a value of max(*C*)/2, which served as a probable midpoint value for the populations’ survivorship.

For the twelve chemical relationships discussed in this paper, the average number of iterations required to identify best-fit parameter values was 168.4. The minimum number of iterations required was 100, which represents the 14 day exposure of dimethyl formamide (dotted lines, Fig 3). The maximum iterations, 299, were required during the fitting procedures for the 7 day exposure of triethylene glycol (solid blue lines, Fig 3). Fitted parameter values for these data are provided in Table 2.

**Table 2:** Parameter values for the curve-fits to sigmoid equations used in Fig 3.

Exposure (days)	Chemical	Reference chemical				Novel chemical			
50.	51.	*V_i_*	*V_f_*	*K_ref_*	*n*	*U_i_*	*U_f_*	*K_novel_*	*m*
7	triethylene glycol	0.998	0.5	39000	2.687	3.	4.	5.	6.
	acetone	7.	8.	9.	10.	0.959	0	3281	5.612
	dimethyl formamide	11.	12.	13.	14.	1	0	5674	10
14	triethylene glycol	0.950	0	13766	10	15.	16.	17.	18.
	acetone	19.	20.	21.	22.	0.948	0.086	3149	7.064
	dimethyl formamide	23.	24.	25.	26.	0.970	0	4048	4.362
21	triethylene glycol	0.908	0	11542	8.924	27.	28.	29.	30.
	acetone	31.	32.	33.	34.	0.920	0	3078	6.068
	dimethyl formamide	35.	36.	37.	38.	0.961	0	2972	3.386
24	triethylene glycol	0.887	0	9769	6.093	39.		40.	41.
	acetone	42.	43.		44.	0.914	0	3001	5.874
	dimethyl formamide	45.	46.	47.	48.	0.900	0	2434	10

Note these parameter values are non-normalized. Units for *K*_*ref*_ and *K*_*novel*_ are *μ*g/L. All other parameters are unitless.

#### Sigmoid-biphasic relationship

A potential example of biphasic survivorship behavior may be found in the case of selenium (Se) toxicity. Many organisms, such as fish, require a certain concentration of Se accumulated in body tissues to survive optimally [29, 30, 31], typically obtained in the wild through diet. Although a dietary Se deficiency has been shown to increase mortality in salmon [32], an excess Se body-burden is also associated with higher mortality in fish [33]. These mortality features at the extremes of deficiency and excess suggests hormesis-like, or biphasic features in the dose-response between Se body-burden and survivorship in fish. We are unaware of a fully characterized dose-response between Se body-burden and whole-fish survivorship; therefore, we will estimate one here from available data, specifically for the purpose of illustrating how our model results can be applied to similar circumstances.

Fish Se body-burden was estimated from literature datasets for Chinook (*Oncorhynchus tshawytscha*) [34] and Atlantic (*Salmo salar*) [32] salmon, which were consistently fed a diet containing elevated Se content, with the Chinook salmon fed two different diets, termed either SLD or SeMet. Fish were consistently fed these diets for approximately 4 weeks, at which time survivorship and dry-weight Se tissue-residue measurements were made.

To employ the Chinook salmon survivorship data (30 days) reported by Hamilton et al. [34], we extrapolated between dietary and tissue residue Se concentrations for both diet types using a linear equation: residue = 0.553 × Se_diet_ + 1.23 *μ*g/g dry weight (SLD diet, *R* ^2^ = 0.9959); and residue = 0.424 × Se_diet_ + 0.947 *μ*g/g dry weight (SeMet diet, *R* ^2^ = 0.9996). Here, Se_diet_ denotes the dietary Se concentrations in *μ*g Se per g dry weight. However, Atlantic salmon survivorship data (28 days) were reported entirely in terms of dietary selenium content. We therefore similarly extrapolated from the Se dietary data in [31] to approximate tissue residues. Because Poston et al. [32] did not report on the relationship between Atlantic salmon dietary intake and tissue concentrations, we used relationships between dietary selenium and tissue residues reported by [35] for Rainbow trout (*Oncorhynchus mykiss*), because of the similar diets used (sodium selenite). These data, however, exhibited a power-law relationship: residue = 3.98 × Se_diet_^0.73^ *μ*g/g dry weight (*R* ^2^ = 0.914).

Given this combined dataset, a similar curve-fitting approach, i.e. the Nelder-Mead simplex method, was used to identify best-fit parameters of the biphasic equation (Eq 4). However, the fitting of the biphasic equation to these data did not depend on the value of the ratio *K* ^+^ / *K* ^-^, in the sense that many such values gave similar values for goodness of fit. Given that the analytical approximations to the biphasic equation requires *K* ^+^ / *K* ^-^>>1 (Appendix A in S1 File), we identified fitted parameters such that *K* ^+^ / *K* ^-^ ≈ 10.

**Fig 4:**
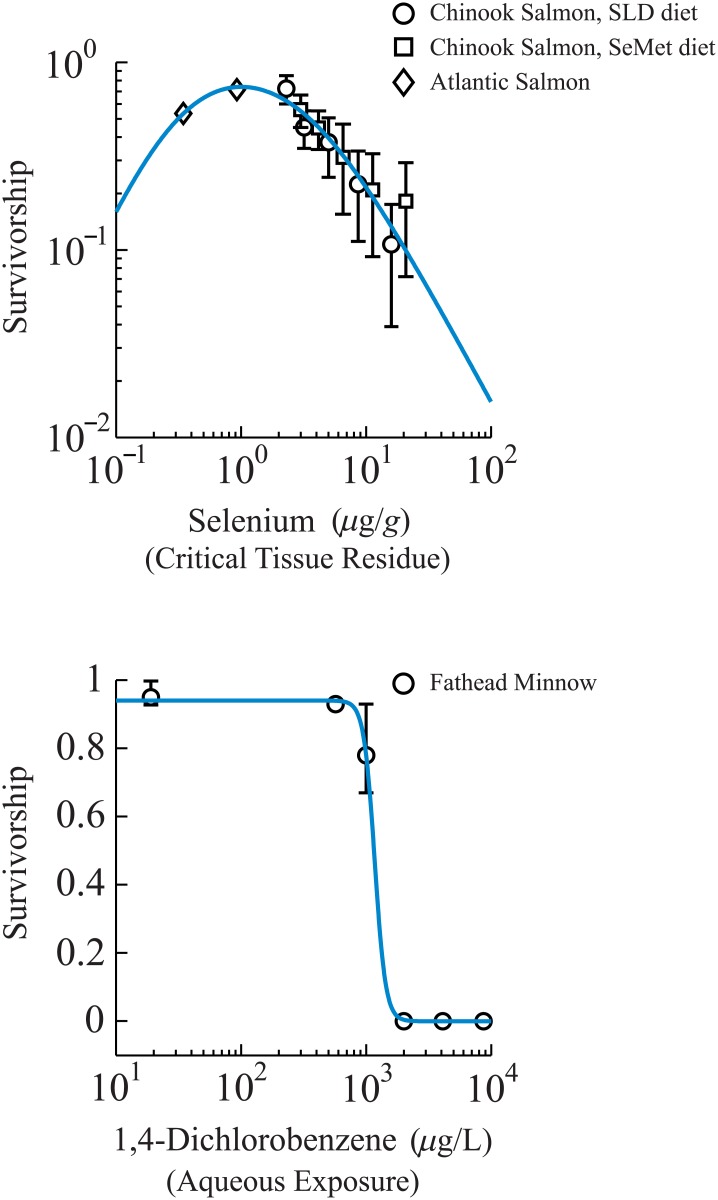
Survivorship data for salmon and fathead minnow. (Top panel) Experimental data illustrating a non-monotone survivorship curve for two species of salmon, *Oncorhynchus tshawytscha* and *Salmo salar* [30, 31], versus Se body-burden measured in *μ*g Se per *g* dry wt tissue. (Bottom panel) A sigmoid survivorship concentration-response curve measuring a cumulative toxic effect for Fathead minnow (*Pimephales promelas*). Data obtained from [33].

To compare these Se data to a survivorship curve based on a commonly used ecotoxicological reference chemical for life-cycle impacts, 1,4-dichlorobenzene [9], we examined survivorship data on another teleost, the Fathead minnow (*Pimephales promelas*) [36]. While using this Fathead minnow data deviates from our requirement that metrics be measured in identical organisms, our goal is to merely demonstrate application of the approach developed in this paper on an exemplary dataset. These Fathead minnow data are shown in Fig 4, which, similar to the *D*. *magna* data in Fig 3, can be seen to closely follow a sigmoid relationship. Parameter values for all empirical fits regarding the sigmoid-biphasic response are given in Table 3. Finally, we emphasize that measurements from aquatic toxicology tests are potentially highly variable; therefore, whether or not Fig 4 reflects a signficant mortality at low Se body-burden in fish will rely on future experiments to increase fidelity between the data and the model, which should settle the issue.

**Table 3:** Parameter values for the sigmoid and biphasic curve fits of Fig 4.

Parameter		Value	Units (description)
1,4-Dichlorobenzene	*V_i_*	0.940	— (population fraction)
	*V_f_*	0	— (population fraction)
	*K_ref_*	1171.5	*μ*g/L (aqueous exposure)
	*n*	9.984	—
Selenium	*U_i_*	0	— (population fraction)
	*U_max_*	1.086	— (population fraction)
	*U_f_*	0	— (population fraction)
	$K_{novel}^{-}$	0.3253	*μ*g/g dry wt (body burden)
	$K_{novel}^{+}$	3.253	*μ*g/g dry wt (body burden)
	*m* ^-^	1.478	—
	*m* ^+^	1.237	—

Note these values are non-normalized. Body burden data have been expressed in units of *μ*g chemical per *g* dry wt tissue.

### Normalization of the concentration-response curves

The toxicity endpoint of survivorship (and therefore mortality) is manifestly a probabilistic measure of a population’s response to toxicant exposure/dosage. The survivorship curve, as given by Figs 3 and 4 (bottom panel), measures the cumulative effect of the population’s response to toxicant exposure. This distribution should, in principle, be normalized, so that the cumulative effect ranges between 0 to 1. In practice, toxicity experiments carried out over chronic-effect timescales allow for population variability stemming from death due to natural causes potentially unrelated to the experimental conditions. To adjust toxicity measurements for significant mortality in control populations, Abbott’s correction [37] and other methods [38] may be applied to population data before any empirical methods are used to estimate the median mortality (LC_50_). However, the control mortality data from our literature-sourced datasets [27,32,34,36] falls within the community-established limit of acceptable loss (<10%). Despite “pre-processing” using these correction methods, it is possible the best-fit cumulative (survivorship/mortality) curves do not begin and end with 0% and 100% (or a fractional response between 0 and 1), respectively, such as shown in Fig 3. One way to address this problem is to fix the endpoints to the desired levels (e.g., to 0 and 1 for Eqs 2 and 3); however, this is an artificial constraint on the response of the population to the toxicant. We therefore seek a method for response-function normalization method which involves manipulating empirical curves that result from unconstrainted empirical fits of the corrected survivorship data. Herein we refer to such a method as *ad hoc* normalization.

A biphasic survivorship curve, however, does not reflect a cumulative effect across the whole of the exposure/dosage range, but rather a combined effect which stems from the underlying biological response to the toxicant in different concentration regimes. For example, Fig 4 (top panel) illustrates one potential example of a biphasic survivorship curve. It is possible there are two regimes of concentration-response here: at lower Se body-burden, survivorship is low but increases monotonically toward a global maximum, such that further increases in body-burden only decrease survivorship monotonically. One explanation for such an effect is if a toxic pathway responds primarily to Se deficiency, but an independent toxic pathway becomes “activated” in response to excess Se bioaccumulation. This is one motivation for decoupling of Eq 5 into two regimes of exposure according to a threshold concentration *C* ^−/+^, with each exposure regime (*C* ≤ *C* ^−/+^ or *C* > *C* ^−/+^) expressing an approximately independent cumulative effect. Thus, each regime for the biphasic response can be separately and independently considered for *ad hoc* normalization, which is self-consistent due to continuity of the concentration-response function at the threshold value.

In the Supporting Information (S1 File), we have provided transformation equations for *ad hoc* normalization of empirical response curves, which aim to rescale the fitted parameters of Eqs 1, 2, and 4, such that the response function resides on a definite fixed interval. This procedure may be used to satisfy probability requirements for the cumulative effect described above (e.g., between 0% and 100%). Consider that concentrations which correspond to a specific response value (e.g., LC_50_), do not change their values upon dilation or contraction of the response axis; therefore, an *ad hoc* transformation of the response axis will not alter the form of the dose-response,

This *ad hoc* normalization procedure is appropriate only for response functions wherein the fractional population responses (e.g., 10%, 50%, 90%) should be equated across different chemicals, which answers the question, “What chemical concentrations elicit an equivalent effect?” For the case of survivorship, the answer to this question may or may not involve *ad hoc* normalization across one or both empirical curves. Hence, extreme care should be taken when considering whether or not to transform the response axis using this (or other) methods. For example, if the resultant effect from an increase (or decrease) in the exposure concentration contributes in an approximately cumulative manner to the response (e.g., sigmoid-like survivorship), and if two concentration-response datasets span a similar response range, then *ad hoc* normalization may be appropriate. However, if responses substantially differ across a similar concentration range, e.g., if response functions saturate at substantially different levels, then it may not be appropriate to normalize across different chemicals despite an identically measured endpoint. For these and other reasons it is important to constrast our model results between the normalized and non-normalized cases.

**Fig 5:**
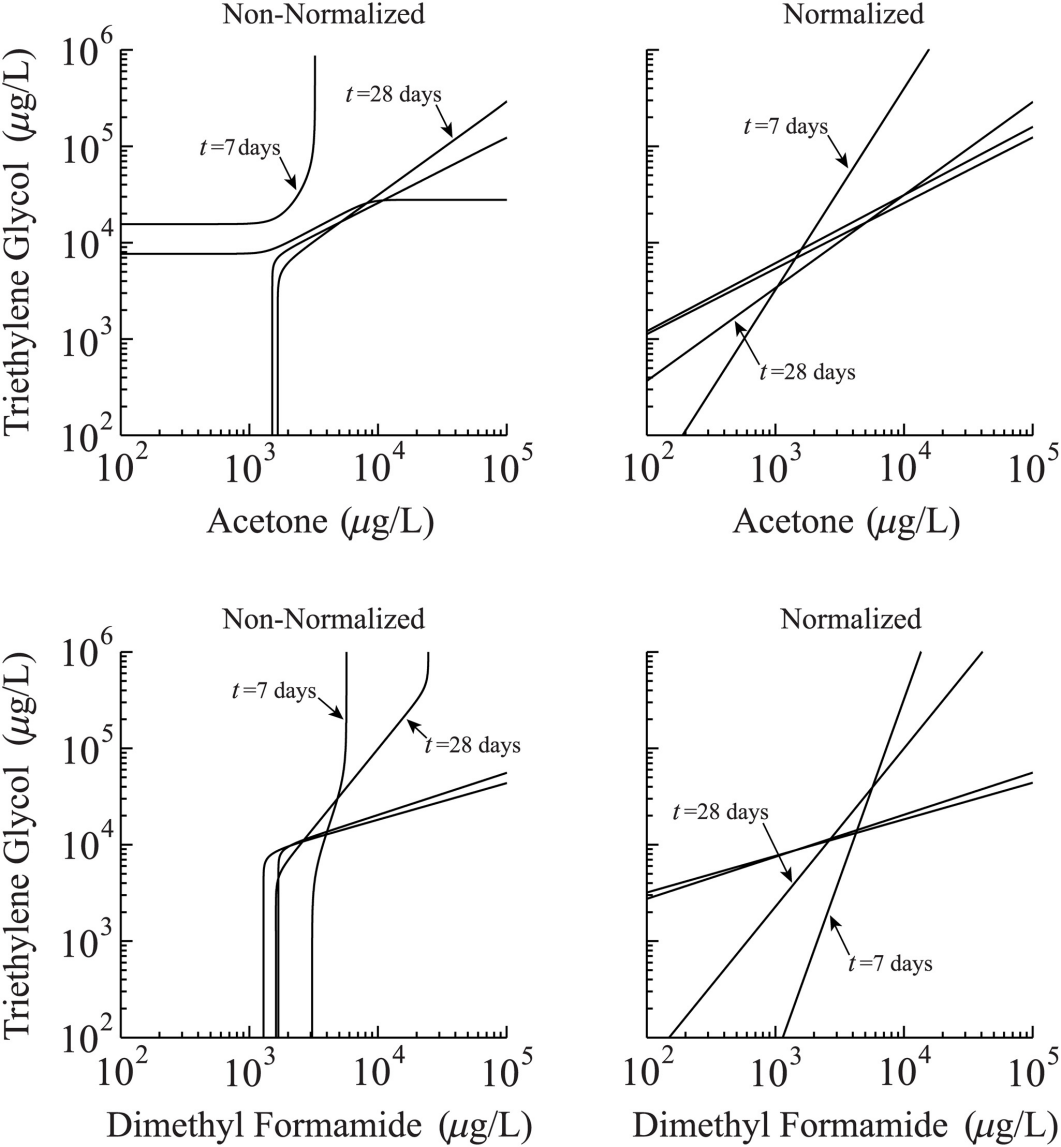
Concentration-concentration relationships derived from sigmoid survivorship data. Comparison between non-normalized (left panels) and normalized (right panels) concentration-concentration response functions derived from the sigmoid data of Fig 3.

Fig 5 contrasts effects between normalized and non-normalized concentration-concentration equivalency relationships expressed using the fitted parameters (Table 2) for the sigmoid-sigmoid data of Fig 3. As evidenced from the non-normalized datasets for acetone (Fig 5, top left panel) and dimethyl formamide (Fig 5, bottom left panel), the concentration-concentration relationship between these chemicals and the reference, triethylene glycol, is undefined below or above certain threshold values, which depend upon the exposure time of the toxicant. This discrepancy is an artifact of the curve-fitting procedure, because, as mentioned above, the survivorship data does not always span identical levels between toxicant treatments. For example, the 7-day line of Fig 5 (top left panel) depicts a non-normalized relationship between triethylene glycol and acetone, despite that an empirical fit to the 7-day triethylene glycol data of Fig 3 (blue line, top left panel) spans from 100% (low concentrations) to approximately 50% (high concentrations). This unusually limited response range may reflect a requirement for more data. Nevertheless, if we take this empirical outcome at face-value, then comparing it directly with the acetone curve (which spans approximately 100% to 0%, Fig 3 top-left panel, blue line) yields a concentration-concentration response with unexpected features (Fig 5, 7-day curve, top-left panel). For example, our model predicts that a single triethylene glycol concentration is ‘equivalent’ to a range of acetone values spanning approximately from 0 to 10^3^ *μ*g/L acetone, indicating these two chemicals are uncorrelated. However, a divergence at approximately 3x10^3^ *μ*g/L acetone indicates the correlation is undefined for higher exposure concentrations. If we instead apply the ad-hoc normalization procedure to the triethylene glycol data, we find a power-law correlation defined for all acetone concentrations.

**Fig 6:**
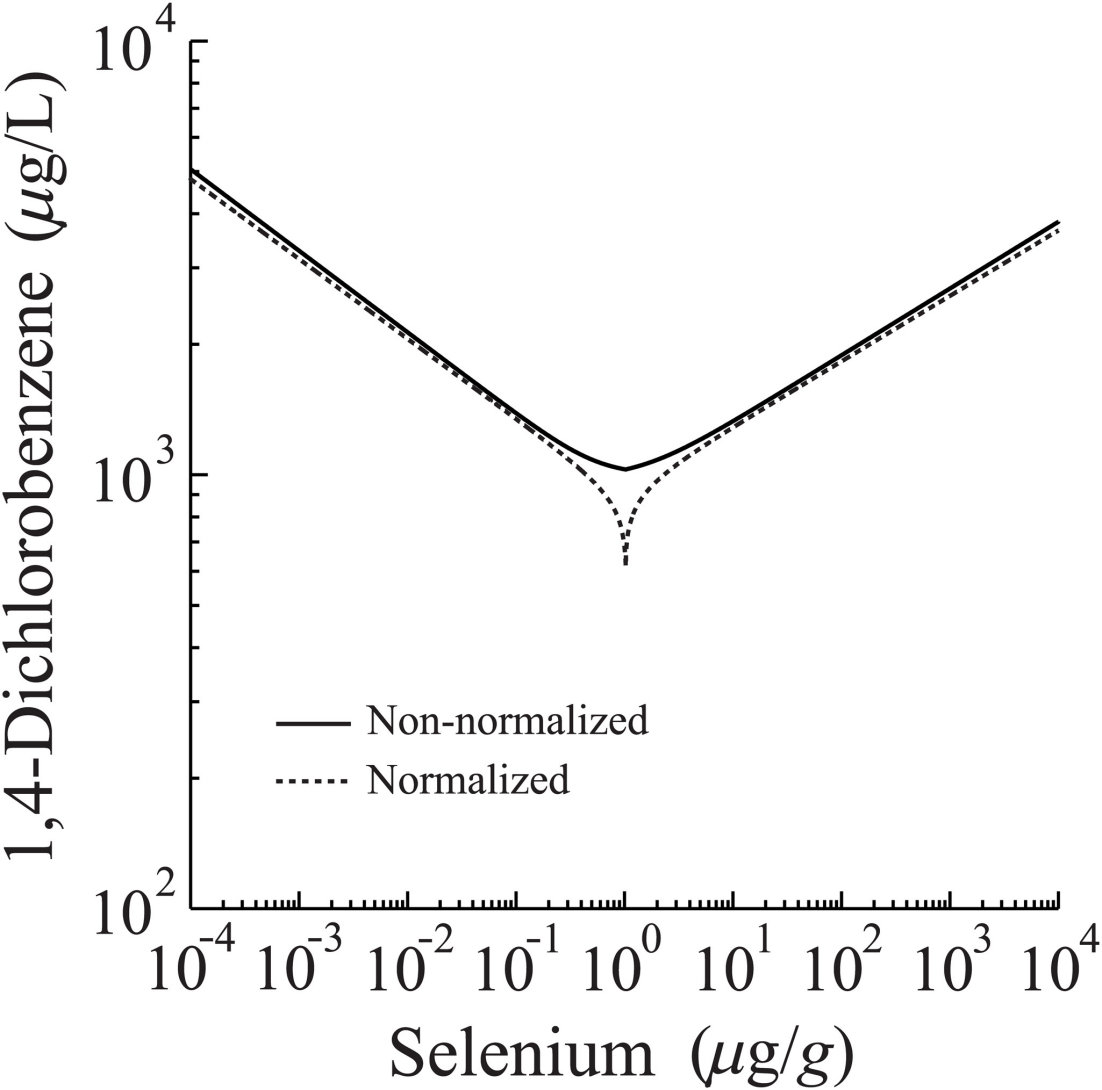
Concentration-concentration relationships derived from sigmoid and biphasic survivorship data. Comparison between non-normalized (solid line) and normalized (dotted line) sigmoid and biphasic concentration-response functions derived from the data of Fig 4.

Fig 6 illustrates results of the normalization procedure for the biphasic dataset of Fig 4 (top panel), and contrasts it against the case without *ad hoc* normalization. Due to the (potential) biphasic nature of the survivorship response, we have decoupled it into a regime of increased survivorship (*C* ≤ *C* ^+/−^ ≈ 1.03*μ*g/L, per Table 3) and a regime of decreased survivorship (*C>*1.03 *μ*g/L). As discussed above, each opposing regime reflects an independent cumulative effect from Se body-burden, which can be modeled individually by sigmoid-like equations (Eq 2). Each such equation (reflecting the novel chemical response) can then be equated with the response of 1,4-Dichlorobenzene (the reference chemical), and the results combined piecewise to approximate the full concentration-concentration relationship, both with (Fig 6, dotted line) and without *ad hoc* normalization (Fig 6, solid line). For either relationship there is little difference in the qualitative response of the concentration-concentration function. For example, they show similar asymptotic behavior. The primary difference between curves resides near the optimal Se concentration, approximately 1.03 *μ*g/L—i.e., any increase or decrease from this concentration value decreases survivorship. However, near this threshold value, the concentration-concentration relationships deviate significantly, which can be directly attributed to the normalization of the local maximum of the biphasic curve. An approximately 1 *μ*g/L selenium concentration coincides with the maximal (normalized) survivorship value, which, according to Fig 4, occurs for a 1,4-Dichlorobenzene concentration of <10^3^ *μ*g/L. The overall result of this normalization-enforced match between the two curves, is that near a 1 *μ*g/L Se concentration, *any* 1,4-Dichlorobenzene concentration value of <10^3^ *μ*g/L gives the equivalent response; hence, the divergence-like behavior observed in Fig 6.

## Results and Discussion

### Validity of the equivalence equations

While Eq 3 provides an exact solution for the sigmoid-to-sigmoid concentration correlation function, the analytic Eqs (5–18), which collectively model the biphasic-to-sigmoid concentration-concentration relationship, are, at best, only approximate solutions. More specifically, approximation enters into this concentration-concentration correlation function through the sigmoid models used to approximate the full biphasic equation, which were proposed to individually model the “lower” and “upper” segments. However, as shown in Figs A and B in S1 File, these sigmoid models exactly match the full biphasic function for *K* ^+^ / *K* ^-^ →∞. This condition is intuitive: if the sigmoid-like positive and negative affectors that compose the biphasic equation were positioned “further apart” by increasing the interval ln*K* ^+^ − ln*K* ^-^ (e.g., Fig B in S1 File), then saturation levels for the positive affector more closely match the starting levels of the negative affector, and in sigmoid models that exhibit very good agreement with the overall biphasic relationship. The relative error of this fitting method is small, even when the positive and negative affectors are positioned closer together; just one example from the Supplemental Information (Fig B in S1 File) gives a maximum (absolute) relative error value of approximately <5.6%.

**Fig 7:**
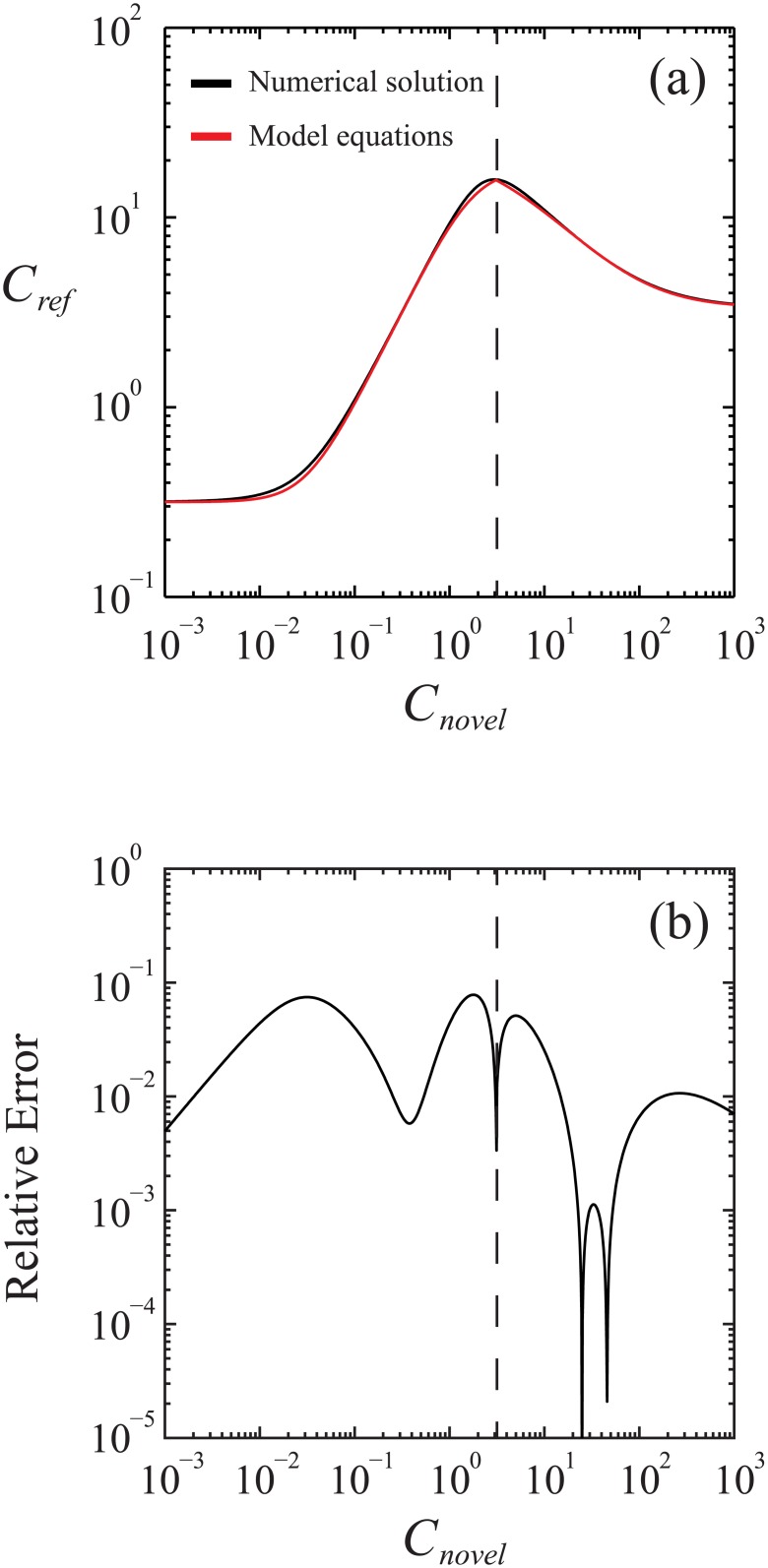
Validity of the sigmoid and biphasic concentration-concentration relationships. (a) Validity of the analytic equations for the concentration-concentration relationship (red line) given by Eqs 5–18 in the main text, overlaid with “exact” numerical results (black line). (b) Absolute value of the relative error between the analytic equations and the exact numerical result.

This approximation of the biphasic response function (Eqs A5 and A6 in Appendix A of the S1 File) manifests in the full concentration-concentration correlation function, and Eqs 5–18 provide a model for this correlation function. Fig 7 overlays this result (Eqs 5–18, red lines) with the exact solution (black line) found numerically using an exemplary parameter set (Table A in S1 File). While the absolute value of the relative error remains small (approximately <7.8%), it is somewhat larger than for the sigmoid-based decomposition of the biphasic equation (see above), quantifying error propagation. As with the sigmoid-based piecewise decomposition of the biphasic response function (see above), Eqs (5–18) become more exact of the concentration-concentration correlation function under conditions of larger ln*K* ^+^ − ln*K* ^-^.

### Chemical equivalence is manifestly nonlinear at low concentrations

One application in which there exists a need for chemical equivalency models is Life Cycle Impact Assessment (LCIA), although there are others, such as in the field of mixtures toxicology [4] and pharmacology [39]. A primary goal of these methods is to estimate the quantity of one chemical concentration in terms of another that give similar effects at the dose-response level.

In the past, formulation of equivalency factors has assumed a linear effects-based relationship between two chemicals. In toxicology, this can sometimes be justified on a mechanistic basis at the molecular level. For example, [25] explains that if a receptor-mediated interaction between a ligand [*L*] and its receptor [*R*], serving as the rate-limiting step of a toxic response, follows the chemical “reaction” [*L*]+[*R*] ↔ [*LR*] to form a complex [*LR*], then it may be responsible for mediating downstream signaling events. Mass-action kinetics gives the steady-state complex concentration as [*LR*]/[*R*]_*total*_ = [*L*]/(*K*_*L*_+[*L*]), wherein [*R*]_*total*_ is the total (fixed) receptor concentration and *K*_*L*_ is the ratio of reverse to forward reaction rates; thus, for “low” concentrations [*L*] << *K*_*L*_, we have: [*LR*]/[*R*]_*total*_ ∼ [*L*]/*K*_*L*_. While this argument may apply when the toxic response depends proportionally on the first step of the toxic pathway (e.g. receptor complex formation), an accounting for downstream- and multiple-binding events in a signal transduction pathway may invalidate the common practice of using a single Hill equation to estimate the input-output properties of the pathway response [40]. In addition, current methods often employ the quasi-steady-state assumption (QSSA) to approximate concentrations of reaction intermediates within a multi-step pathway, despite that reactants may be present at comparable concentrations [41], or when decoupling intermediate dynamics based on time-scales is inappropriate [42].

To determine the conditions on parameter values and concentrations wherein the equivalence relationship is approximately linear, we will employ the sigmoid-sigmoid equivalence relation of Eq 3 above; we need not separately consider the biphasic relationship, because Eqs 5–18 were likewise expressed in terms of similar equations to Eq 3, but with different parameter values. To proceed, we assume that both sigmoid-based dose-response curves are normalized, consistent with a practice of measuring relative, versus absolute, median-effect concentrations (e.g. EC_50_) for some toxicity endpoints. Each curve therefore begins and ends at identical response levels over the breadth of the concentration range. For Eq 3, normalization means that *U*_*i*_ = *V*_*i*_ and *U*_*f*_ = *V*_*f*_, which greatly simplifies the concentration-concentration equation:

$$\text{Ex:}\;C_{ref}=K_{ref}{\left[\frac{C_{novel}}{K_{novel}}\right]}^{m/n}.$$ (20)

To examine how *C*_*ref*_ behaves at small concentrations, $C_{novel}=C_{novel}^{0}\approx 0$, we can expand Eq 20) in a Taylor series about this value, representative of the low concentrations typical of environmental contamination:

$$\text{Ex:}\;C_{ref}=K_{ref}{\left(\frac{C_{novel}^{0}}{K_{novel}}\right)}^{m/n}\left[1+\frac{m}{n}\left(\frac{C_{novel}}{C_{novel}^{0}}-1\right)+\frac{m}{n}\frac{m-n}{2n}{\left(\frac{C_{novel}}{C_{novel}^{0}}-1\right)}^{2}+\vartheta {\left(\frac{C_{novel}}{C_{novel}^{0}}-1\right)}^{3}\right].$$ (21)

Here, $\vartheta {\left(\frac{C_{novel}}{C_{novel}^{0}}-1\right)}^{3}$ denotes terms in the series of order ≥ 3. In order for the linear term to dominate this series, the second-order term must be smaller than the first two terms, the third-order term must be smaller than the preceding three terms, and so on. We can therefore estimate this threshold by comparing absolute values of the first- and second-order terms:

$$\frac{m\left|m-n\right|}{2n^{2}}{\left(\frac{C_{novel}}{C_{novel}^{0}}-1\right)}^{2}<<\frac{m}{n}\left(\frac{C_{novel}}{C_{novel}^{0}}-1\right)$$

which can be manipulated to give:

$$\text{Ex:}\;\frac{C_{novel}}{C_{novel}^{0}}<<1+2\frac{n}{\left|m-n\right|}$$ (22)

Note that the right-hand side of Eq 22) diverges for *m = n*, indicating that the concentration-concentration equation is linear for any concentration value. Indeed, this can be checked by putting *m = n* into Eq 20).

Threshold (22) can be made more intuitive by noting how the slopes of the sigmoid dose-response curve relate to the parameters *m* and *n*, by noting that the slope of a sigmoid is maximal at its inflection point. In the terminology of Eq 1, the slope of the sigmoid on a log-log curve at the inflection point is the exponent of a power-law, which can be given by the equation:

$${\text{slope}}_{ref}=n\frac{\sqrt{V_{f}}-\sqrt{V_{i}}}{\sqrt{V_{f}}+\sqrt{V_{i}}}$$

A similar equation exists for the logarithmic slope for the concentration-response of the novel compound at its inflection point. Because we have assumed that the initial and final levels of the response profiles are normalized, Eq 22) can be recast into a more intuitive form, expressed in terms of the logarithmic slopes:

$$\text{Ex:}\;\frac{C_{novel}}{C_{novel}^{0}}<<1+2\frac{{\text{slope}}_{ref}}{\left|{\text{slope}}_{novel}-{\text{slope}}_{ref}\right|}.$$ (23)

Thus, we find that when the slopes are equal, Eq 23) diverges, which menas that Eq 2 is linear everywhere.

Restriction of chemical equivalence to only “parallel” concentration-response functions has been noted before [4] as a necessary condition of the toxic equivalency factor (TEF) method employed in the field of mixtures toxicity. However, we have here formalized and validated this requirement mathematically (Eqs 22 and 23).

Finally, we point out that that Eq 20) is nonlinear across the entirety of its concentration domain, even for “small” concentrations. This nonlinear property takes the form of a power-law relationship; although, linearity over the whole of the concentration range can be recovered if the fitted parameters of the novel and reference concentration-response functions satisfy conditions (22–23). Thus, a scenario of unequal slopes between two (or more) concentration-response functions is the more likely case, and any linear extrapolation must therefore make reference to a specific (nonzero) concentration along the curve to contrive a linear form from Eqs (3), (5) and (6). This stands in contrast to contemporary equivalence calculations, which rely on the premise that a marginal change in the equivalency relationship is extensive to the whole of the concentration domain [10].

### Calculating an Equivalency Factor

Eq (20) can be used to estimate a value for an Equivalency Factor used in LCIA ecotoxicological impact categories (e.g. such as in [8] or [9]), which is a similar concept to the TEF proposed for mixtures toxicology calculations [4]. Within an LCA context, the equivalence factor, *EF*, is defined as the derivative of the concentration-concentration relationship evaluated at a fixed exposure/environmental concentration, $C_{novel}^{\text{exp}}$, of the novel compound:

$$\text{Ex:}\;EF=\left(\frac{dC_{ref}}{dC_{novel}}\right)\left(C_{novel}=C_{novel}^{\text{exp}}\right)=\frac{m}{n}\frac{{\text{EC}}_{\text{50}}^{\text{ref}}}{{\text{EC}}_{\text{50}}^{\text{novel}}}{\left(\frac{C_{novel}^{\text{exp}}}{{\text{EC}}_{\text{50}}^{\text{novel}}}\right)}^{m/n-1}.$$ (24)

Here we have labeled $K_{ref}={\text{EC}}_{\text{50}}^{\text{ref}}$ and $K_{novel}={\text{EC}}_{\text{50}}^{\text{novel}}$, as median-effect concentrations of a test population, or generally the half-maximal effect of the (normalized) response, assuming a situation wherein normalization is appropriate between response functions. A special case of Eq 24) involves concentration-response curves that are “parallel,” i.e. *m = n*, then Eq 24) reduces to:

$$\text{Ex:}\;EF=\frac{{\text{EC}}_{\text{50}}^{\text{ref}}}{{\text{EC}}_{\text{50}}^{\text{novel}}}.$$ (25)

This result is independent of any concentration value, which is similar to previous results obtained using different methods [9, 43, 44].

### Uncertainty within LCA Studies

Finally, it must be noted that with respect to LCA as a methodology, equivalence modeling is but one step in a lengthy series of mathematical processes. Any of these processes can be an entry point for uncertainty to then further propagate [19]. For instance, in developing a characterization factor, constituent factors representing environmental fate, effects, and chemical equivalence are typically constructed. Mayo et al. [45] found that a commonly used fate and transport model for LCA studies was highly sensitive to parameter value fluctuations, varying up to 8 decades in magnitude, consistent with findings in other similar models [46, 47]. These variances can further propagate through the phases of the Interpretation stage, resulting in potentially unreliable and misleading results.

This represents a problem for LCA, which ultimately serves as a decision aid to inform some type of future management action (e.g., to enhance the sustainability of manufacturing processes). Based on the concept of decision quality [48], if the information about the decision is unreliable, then the resulting decision may not be as good as it could be if the information were of higher quality. Thus, the underlying goal in improving upon the methods in the chemical equivalency model, as with efforts aimed at improving other phases of the LCA, is to provide the decision maker with meaningful, reliable information which can then be translated into effective environmental management actions [49].

## Conclusions

We have proposed a method for calculating concentration-concentration relationships between two chemicals parameterized by an experimental endpoint, such as survivorship or mortality metrics from toxicology. Our proposed method is general, and can be applied to many other measured biological endpoints, such as enzyme activity or metabolite concentrations, among others. Working from the perspective and terminology of toxicology, we derived equivalence relationships for two types of concentration-response curves: sigmoid, and biphasic or “U-shaped” curves.

While our equations for the sigmoid-sigmoid response are exact, we provided analytical equations for the sigmoid-biphasic relationship that closely approximated the full concentration-concentration curves obtained numerically. In both cases these equivalence relationships were found to be manifestly nonlinear, following the general form of a power-law, which we exemplified with experimental datasets obtained from the toxicology literature. Such nonlinearity persists even at the environmentally relevant regime of low concentration, and can only be ameliorated by a linear extrapolation from specific points along the concentration-response curve. Thus, current methods that seek to linearize concentration-response functions, even at low concentrations, are inappropriate when constructing equivalence relationships expected as valid everywhere.

To address this problem, we used our concentration-concentration response functions to derive an expression for an equivalence factor employed in many LCIA impact characterization models. This calculation is also representative of methods proposed previously for use in mixtures toxicology, and the pharmacology of drug interactions. We found that popular equivalency factor constructions that simply compute the ratio of EC_50_ values (or LC_50_, or AC_50_, etc.) of reference to novel chemicals, may only be used if: (i) the concentration-response relationships between chemicals are normalized to identical initial and final concentrations; and (ii) if the concentration-response functions are “parallel,” which we formalized in terms of mathematical conditions on the ratio of two fitted parameter values of our models. In the TEF approach to mixtures toxicology explained by Safe [4], these conditions were mandated, but not formalized using quantifiable relationships. We have remedied this problem for ecotoxicological concentration-response functions, by providing conditions on the curve-fitted sigmoid or biphasic parameter values needed to validate this approach.

Among the mathematical models and methods that employ equivalence relationships, LCA serves a primary role in estimating environmental impacts associated with human activity, and thus supports environmental decision-making aimed at mitigating impacts, and promoting sustainability. While formal and site-specific risk assessments can be conducted *after* a contaminant is released into the environment, the LCA framework is uniquely future-oriented. Attributed to this predictive nature is uncertainty inherent within its results, which stems from many sources including its reliance on noisy experimental data and in the compounding nature of its modeling methods, such as in many impact characterization models. However, the fidelity of LCIA results could potentially be improved through inclusion of data-driven nonlinear representations of chemical equivalence described in this paper, due to its realistic treatment that minimizes use of broad approximation.

It is important to note that applications of our approach are not strictly limited to existing methods in mixtures toxicology, or to existing LCIA calculations: any experimental metric which exhibits a concentration-response function can leverage our approach and its results. For example, other LCIA impact categories equally require extrapolation from one chemical contaminant to a common reference material, such as with the global warming (e.g. CO_2_-equivalents), acidification (SO_2_-equivalents), or eutrophication (PO_4_-equivalents) impact categories (Table 1). Further research will be needed to fully develop these and other equivalency models. Moreover, analyses where it may be convenient to represent one chemical in terms of another, including risk and hazard assessment, and most of the environmental sciences, could potentially utilize the methods described herein.

## Supporting Information

S1 File *Derivation of Biphasic Model Equations and Response Function Normalization Methods*. The Supporting Information includes derivations of equations for analytic approximations to the biphasic response function in terms of model sigmoid equations (Appendix A). In addition, transformation equations are given for parameter values that enforce a normalization between sigmoid and biphasic concentration-response functions (Appendix B). Fig A illustrates the sigmoid-like components of the positive and negative affectors composing the biphasic response function. Fig B illustrates the relative error between the sigmoid-like approximations for the left- and right-hand sides of the biphasic response and the full biphasic response. Fig C conceptualizes the ad hoc normalization method. Fig D illustrates how the sigmoid and biphasic response functions could be compared. Table A provides parameter values for the plots shown in Fig B.
(PDF)
