# Correction: Data-Driven Method to Estimate Nonlinear Chemical Equivalence

**DOI:** 10.1371/journal.pone.0134652

**Published:** 2015-07-30

**Authors:** The PLOS ONE Staff

In the Methods section, there is an error in equation six in the section titled “Equation for chemical equivalence: sigmoid-biphasic response functions.” The greater-than sign on the right side of the equation was incorrectly replaced with a less than or equal to sign. The publisher apologizes for this error. Please see the complete, correct equation here:

$$C_{ref}=K_{ref}{\left[\frac{\left(U_{f}-V_{i}\right){\left(C_{novel}/{\tilde{K}}_{novel}^{+}\right)}^{\tilde{m}+}+{\tilde{U}}_{\text{max}}^{eff,+}-V_{i}}{\left(-U_{f}+V_{f}\right){\left(C_{novel}/{\tilde{K}}_{novel}^{+}\right)}^{\tilde{m}+}-{\tilde{U}}_{\text{max}}^{eff,+}+V_{f}}\right]}^{1/n},\text{ for }C_{novel}>C_{novel}^{-/+}.$$ (6)
