# Supplementary material for: Data-Driven Method to Estimate Nonlinear Chemical Equivalence
Source: PLoS One. 2015 Jul 9;10(7):e0130494. doi: 10.1371/journal.pone.0130494 (PMC4497723; doi:10.1371/journal.pone.0130494)
Supplement: S1 File — The Supporting Information includes derivations of equations for analytic approximations to the biphasic response function in terms of model sigmoid equations (Appendix A). In addition, transformation equations are given for parameter values that enforce a normalization between sigmoid and biphasic concentration-response functions (Appendix B). Fig A illustrates the sigmoid-like components of the positive and negative affectors composing the biphasic response function. Fig B illustrates the relative error between the sigmoid-like approximations for the left- and right-hand sides of the biphasic response and the full biphasic response. Fig C conceptualizes the ad hoc normalization method. Fig D illustrates how the sigmoid and biphasic response functions could be compared. Table A provides parameter values for the plots shown in Fig B. (PDF) [file pone.0130494.s001.pdf]

Supporting Information: File S1

Data-driven method to estimate nonlinear chemical equivalence

Michael Mayo[1], Zachary A. Collier[1], Corey Winton[2], Mark A. Chappell[1]

[1]Environmental Laboratory, US Army Engineer Research and Development Center, Vicksburg, MS 39183;

[2]Information Technology Laboratory, US Army Engineer Research and Development Center, Vicksburg, MS 39183;

Appendix A:

Derivation of Biphasic Model Equations

Equations for the biphasic-sigmoid concentration function

We obtain the concentration-concentration correlation for a reference chemical that exhibits a sigmoid dose-response curve, and a "novel" chemical that exhibits a biphasic dose-response curve, which can be modeled using the equation:

$$f(C) = \frac{\left[1+\left(C/K^-\right)^{m^-}\right]\left[U_{\max}+U_f\left(C/K^+\right)^{m^+}\right]-U_{\max}+U_i}{\left[1+\left(C/K^-\right)^{m-}\right]\left[1+\left(C/K^+\right)^{m^+}\right]}. \quad \text{(A1)}$$

Here, C is the "novel" chemical concentration; K^- and K^- are concentrations that give half of the maximum concentration, $U_{\max}$, for each of the respective constituent sigmoids; $\tilde{m}^-$ and $\tilde{m}^+$ are parameters that help to set the slope of the constituent sigmoids at their respective inflection points; and U_i and U_f are the response values at the respective concentration extremes, $C = 0$ and $C = \infty$. Note that we have dropped the term "novel" from the subscript of the parameters, which is otherwise present in the main text; there is no need to distinguish between "novel" or "reference" chemicals in the calculations described here.

As explained in the main text, the dose-response function, $f(C)$, must be obtained for both reference and novel chemicals from identical experiments (e.g., Mortality measured in Fathead minnow). This restriction allows for the response of the dose-response function to be used as a parameterization variable of the concentration-concentration correlation function. Unlike the sigmoid-sigmoid relationship obtained in

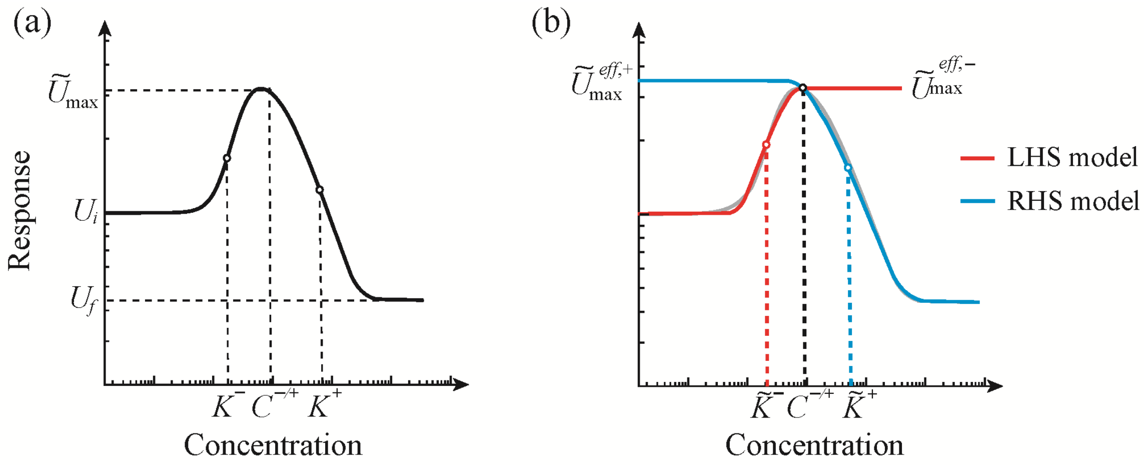

Figure A. (a) Example of a biphasic dose-response. (b) Sigmoid equations can be used to model the left- (red line) and right-hand (blue line) sides of the biphasic response.

the main text, the biphasic function generally yields two outputs for one input, or vice versa, depending on the choice of reference and novel chemical.

To begin, we first assume that the biphasic and sigmoid functions have been fitted to dose-response data so that parameter values are given. The goal will be to use parameter values of Eq. (A1), and nothing else, to find analytic equations that estimate the concentration-concentration correlation function.

Partitioning the biphasic function using a threshold novel chemical concentration

We first partition the biphasic function into parts that individually yield one-to-one correlation functions. Depending on the fitted parameter values of the biphasic function to the dataset, it may exhibit either a local maxima or minima. Here we consider exemplary parameters that provide a local maximum, as shown in Fig. A; although, the equations we derive may be applied to the case of a local minimum. We partition the biphasic curve into halves, depending on whether the novel chemical concentration falls above or below at a threshold value, $C^{-/+}$, which is presented in the main text:

$$C^{-/+} = \left[K^{-}\left(K^{+}\right)^{(\,\cdot\,)}\right]^{1/[1+(\,\cdot\,)]} \left\{ \sqrt{\frac{U_i}{U_{\max}}}^{1/m^{-}} \left[\sqrt{\frac{U_{\max}}{U_f}} \left(\sqrt{\frac{U_f}{U_{\max}}} \cdot \sqrt{\frac{U_{\max}}{U_i}} \right)^{\frac{1+\sqrt{U_f/U_{\max}}}{1-\sqrt{U_f/U_{\max}}}} \right]^{(\,\cdot\,)/m^{+}} \right\}^{1/[1+(\,\cdot\,)]} , \tag{A2}$$

wherein we have abbreviated:

$$(\,\cdot\,) = \frac{m^{+}}{m^{-}} \cdot \frac{\dfrac{1-\sqrt{U_i/U_{\max}} \cdot \sqrt{U_f/U_{\max}}}{\sqrt{U_f/U_{\max}} - \sqrt{U_i/U_{\max}}} - 1}{\dfrac{1-\sqrt{U_i/U_{\max}} \cdot \sqrt{U_f/U_{\max}}}{\sqrt{U_f/U_{\max}} - \sqrt{U_i/U_{\max}}} + 1} . \tag{A3}$$

Equations (A2) and (A3) may also be approximated with the geometric mean of the parameters K^{+} and K^{-}:

$$C^{-/+} \approx \sqrt{K^{-}K^{+}} . \tag{A4}$$

Component models for the decomposed biphasic curve

As given by Eq. (A4), we partition the biphasic curve into two pieces, referred to herein as its left-hand (LHS) and right-hand (RHS) sides. We model each such portion with a sigmoid equation, which are, in principle, different than the constituent sigmoid-like equations used in construction of the biphasic curve equation. Parameters associated with the LHS sigmoid are herein denoted by a minus sign, -, while parameters of the sigmoid modeling the RHS of the biphasic function are herein denoted using an addition sign, +. Thus, sigmoid equations that model the LHS and RHS are respectively given by:

$$f^{-}(C_{novel}) = U_i + \left(\tilde{U}_{\max}^{eff,-} - U_i\right)\frac{\left(C_{novel}/\tilde{K}^{-}\right)^{\tilde{m}-}}{1+\left(C_{novel}/\tilde{K}^{-}\right)^{\tilde{m}-}}$$, for $C_{novel} \le C^{-/+}$ (LHS), and (A5)

$$f^{+}(C_{novel}) = \tilde{U}_{\max}^{eff,+} + \left(U_f - \tilde{U}_{\max}^{eff,+}\right)\frac{\left(C_{novel}/\tilde{K}^{+}\right)^{\tilde{m}^{+}}}{1+\left(C_{novel}/\tilde{K}^{+}\right)^{\tilde{m}^{+}}}$$, for $C_{novel} > C^{-/+}$ (RHS). (A6)

Values for the parameters U_i and U_f are directly preserved from the fitted parameters of the biphasic response function, because the sigmoid equations modeling the LHS, Eq. (A5), and the RHS, Eq. (A6), must, respectively, coincide asymptotically with the biphasic curve (i.e., at $C_{novel} = 0$ for the LHS, and $C_{novel} = \infty$ for the RHS). Because these sigmoid models are approximate of the biphasic curve, parameters of Eqns. (A5)-(A6) denoted with tildes can be written exclusively in terms of the fitted parameters of the biphasic curve. In the next section we explain how to achieve this for the LHS sigmoid, Eq. (A5). The procedure to obtain effective parameters for the RHS, Eq. (A6), is similar, but yields a different result.

Effective parameters for the LHS of the biphasic curve, Eq. (A5)

Equation (A5) contains three effective parameters: $\tilde{m}^{-}$, $\tilde{K}^{-}$, and $\tilde{U}_{\max}^{eff,-}$. Our general strategy will be to identify conditions that restrict the model sigmoid (e.g., Eq. (A5)) to "match" the response of the biphasic curve in the LHS domain ($C_{novel} \le C^{-/+}$). Because there are three effective parameters, we must obtain three independent conditions, as we explain in the following subsections. We note that these conceptual restrictions are identical for the RHS model sigmoid; the actual form of the equations differ, which leads to slightly different results to those obtained for the LHS sigmoid. Finally, it will be useful

to express the approximate value of the local maximum (or minimum) of the biphasic curve, $\tilde{U}_{\max}$:

$$\tilde{U}_{\max} = \frac{\left(1+\sqrt{K^+/K^-}^{\,m^-}\right)\left(U_{\max}+U_f\sqrt{K^-/K^+}^{\,m^+}\right)-U_{\max}+U_i}{\left(1+\sqrt{K^+/K^-}^{\,m^-}\right)\left(1+\sqrt{K^-/K^+}^{\,m^+}\right)}, \tag{A7}$$

which can be found by evaluating Eq. (A1) at the point $C = C^{-/+} = \sqrt{K^-K^+}$.

Effective parameter: the condition for $\tilde{m}^-$

The parameter $\tilde{m}^-$ sets the slope of the sigmoid model in the log-log scale, evaluated at its inflection point. If the sigmoid model (red line, Fig. A(b)) is to converge with the left-hand side of the biphasic response, then both slopes should be evaluated at their respective inflection points, and should necessarily match in value. Carrying out this calculation gives:

$$\tilde{m}^- = m^- \times \frac{\sqrt{\tilde{U}_{\max}^{eff,-}/Ui}+1}{\sqrt{\tilde{U}_{\max}^{eff,-}/Ui}-1} \times \frac{\sqrt{U_{\max}/Ui}-1}{\sqrt{U_{\max}/Ui}+1}. \tag{A8}$$

Effective parameter: the condition for $\tilde{K}^-$

The previous section applied the restriction that the slopes for the model sigmoid (Eq. (A5)) and that of the left-hand side of the biphasic response should match, which gave an equation for $\tilde{m}^-$ (Eq. (A8)). We now make another restriction: that response values for the model sigmoid and the biphasic response should match, at least once between the initial and final levels of the response function; namely at the point $\sqrt{\tilde{U}_{\max}U_i}$, which

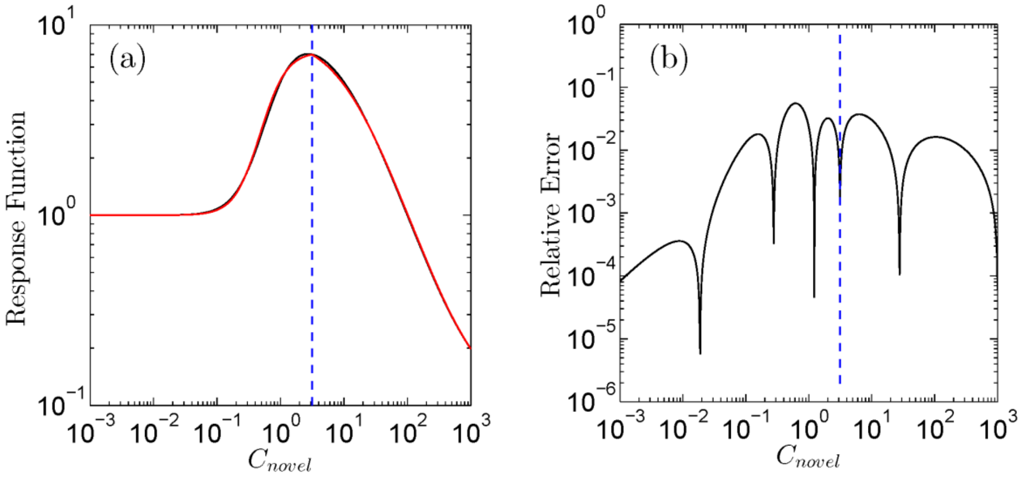

Figure B. (a) Analytic approximation (red lines) for the left-hand (LHS) and right-hand (RHS) sides—delineated by the vertical dotted line given by Eq. (A4)—of the biphasic response function (solid black lines). (b) Absolute values of the relative error calculated between the empirical equation for the biphasic response and the analytic equations of both the LHS and RHS. For these parameters the maximum relative error is approximately 5.6%. Parameter values used for this example have been collected into Table S1.

is the geometric mean between the initial and maximum levels of the left-hand side biphasic response. We can first identify a concentration value that provides this particular value of the biphasic response, denoted by C_{K^-}, when put into the constituent sigmoid of the left-hand side response:

$$\underbrace{U_i + \left(U_{\max} - U_i\right)\frac{\left(C_{K^-}/K^-\right)^{m-}}{1+\left(C_{K^-}/K^-\right)^{m-}}}_{\substack{\text{left-hand side sigmoid} \\ \text{for the biphaisc response}}} = \underbrace{\sqrt{\tilde{U}_{\max}U_i}}_{\substack{\text{geometric mean} \\ \text{between initial and} \\ \text{final levels of the biphasic} \\ \text{response function}}} \quad ,$$

which can be solved, to give:

$$C_{K^-} = K^-\left(\frac{\sqrt{\tilde{U}_{\max}/U_i} - 1}{U_{\max}/U_i - \sqrt{\tilde{U}_{\max}/U_i}}\right)^{1/m^-} .$$

Now, we can ask a similar question: What value for the constant $\tilde{K}^-$ must we pick that will also give the response value $\sqrt{\tilde{U}_{\max}U_i}$ for the model sigmoid (Eq. (A5)), when evaluated at C_{K^-}? Setting this question up mathematically result in the equation:

$$U_i + \left(\tilde{U}_{\max}^{eff,-} - U_i\right)\frac{\left(\tilde{K}^-/C_{K^-}\right)^{\tilde{m}^-}}{1+\left(\tilde{K}^-/C_{K^-}\right)^{\tilde{m}^-}} = \sqrt{\tilde{U}_{\max}U_i} .$$

Solve this equation for $\tilde{K}^-$ gives:

$$\tilde{K}^- = K^-\left(\frac{\sqrt{\tilde{U}_{\max}/U_i} - 1}{U_{\max}/U_i - \sqrt{\tilde{U}_{\max}/U_i}}\right)^{1/m^-}\left(\frac{\tilde{U}_{\max}^{eff,-}/U_i - \sqrt{\tilde{U}_{\max}/U_i}}{\sqrt{\tilde{U}_{\max}/U_i} - 1}\right)^{1/\tilde{m}^-} . \qquad \text{(A9)}$$

Effective parameter: the condition for $\tilde{U}_{\max}^{eff,-}$

Equations (A8) and (A9) both depend on the apparent saturation value, $\tilde{U}_{\max}^{eff,-}$ ($C = \infty$), for the model left-hand side sigmoid (Eq. (A5)). To identify this value, we demand that the model sigmoid of Eq. (A5) match the approximate maximum value of the biphasic response, $\tilde{U}_{\max}$, which occurs at the concentration value: $\sqrt{K^-K^+}$. Thus, we put Eqns. (A8) and (A9) into Eq. (A5): $f^-\left(C = \sqrt{K^-K^+}\right) = \tilde{U}_{\max}$, which results in a complicated equation restricting the value of $X = \tilde{U}_{\max}^{eff,-}/U_i$:

$$\frac{X-1}{1+\left(\frac{X-1}{\sqrt{\tilde{U}_{\max}/U_i}}-1\right)\left\{\left[\sqrt{\frac{K^-}{K^+}}^{m^-}\left(\frac{\sqrt{\tilde{U}_{\max}/U_i}-1}{U_{\max}/U_i-\sqrt{\tilde{U}_{\max}/U_i}}\right)\right]^{\frac{\sqrt{U_{\max}/U_i}-1}{\sqrt{U_{\max}/U_i}+1}}\right\}^{\frac{\sqrt{X}-1}{\sqrt{X}+1}}}=\frac{\tilde{U}_{\max}}{U_i}-1. \qquad \text{(A10)}$$

We propose to solve Eq. (A10) approximately, by noting that the local maximum (or minimum) value of the biphasic function, $\tilde{U}_{\max}$, should be reasonably close to its solution. This suggests that we expand the left-hand side using a Taylor series to first order in a logarithm scale, due to the underlying sigmoid structure. Solving the resulting approximate equation gives:

$$\tilde{U}_{\max}^{eff,-}=\tilde{U}_{\max}\left(1+\chi^-\sqrt{\frac{\tilde{U}_{\max}}{U_i}}\right)^{1/\mu^-}, \qquad \text{(A11)}$$

wherein the variables χ^- and μ^- are, respectively, a prefactor and the exponent of the power-law approximation:

$$\chi^-=\left[\sqrt{\frac{K^-}{K^+}}^{m^-}\left(\frac{\sqrt{\tilde{U}_{\max}/U_i}-1}{U_{\max}/U_i-\sqrt{\tilde{U}_{\max}/U_i}}\right)\right]^{\frac{\sqrt{U_{\max}/U_i}-1}{\sqrt{U_{\max}/U_i}+1}\times\frac{\sqrt{\tilde{U}_{\max}/U_i}+1}{\sqrt{\tilde{U}_{\max}/U_i}-1}}, \text{ and} \qquad \text{(A12)}$$

$$\mu^-=\frac{\tilde{U}_{\max}/U_i}{\tilde{U}_{\max}/U_i-1}\times\frac{\chi^-\left(\ln\chi^--1\right)+1}{1+\chi^-\sqrt{\tilde{U}_{\max}/U_i}}. \qquad \text{(A13)}$$

Effective parameters for the RHS of the biphasic curve, Eq. (A6)

Parameters for the right-hand side sigmoid model, Eq. (A6), of the biphasic response function, Eq. (A1), can be obtained using similar restrictions to each of the three variables described above in the previous section. However, we should note that these

restrictions result in slightly different expressions for the effective parameters $\tilde{m}^+$, $\tilde{K}^+$, and $\tilde{U}_{\max}^{eff,+}$, than are given, respectively, by Eqns. (A8), (A9), and (A11)-(A13).

Validity of the sigmoid-based approximate model to the biphasic response function

Figure B compares the sigmoid models of Eqns. (A5)-(A6) with the "exact" biphasic equation, Eq. (A1), with parameter values given in Table A. As shown in Fig. B(a), the sigmoid models of Eqns. (A5)-(A6) are qualitatively very close to the response of the biphasic function, with a maximum relative error of approximately 5.6% (Fig. B(b)) for the illustrated parameter values. The absolute value of this relative error rises slightly as $K^+/K^- \to 1$ (from above); however, the sigmoid models for both left- and right-hand sides match the biphasic response exactly in the limit $K^+/K \to \infty$, but the match is nevertheless very close for $K^+/K^- >> 1$ (e.g., Fig. B employs $K^+/K^- = 10$).

Finally, we note that, in the above analyses, we have assumed that $U_i > 0$ and $U_f > 0$, as shown in Fig. A. If either or both of these conditions fail (e.g., $U_i = U_f = 0$), then the defining biphasic equation, Eq. (A1), can be simply shifted vertically by an arbitrary amount, such as unity; therefore, only the parameters U_i, U_f, and $U_{\max}$ are affected. This can be shown as follows. First, consider that Eq. (A1) is a composite of two sigmoids; one sigmoid for the positive affector, termed A_{pos}, and another for the negative affector, termed A_{neg}. Now, the negative affector serves as the amplitude for the positive affector curve, so that Eq. (A1) may be obtained by function composition: $f = A_{pos} \circ A_{neg}$. Writing the positive affector as,

$$A_{pos}(x) = U_i + (x - U_i)\frac{(C/K^-)^{m^-}}{1+(C/K^-)^{m^-}},$$

can be manipulated to yield:

$$A_{pos}(x)+1 = (U_i+1)+[(x+1)-(U_i+1)]\frac{(C/K^-)^{m^-}}{1+(C/K^-)^{m^-}}. \quad \text{(A14)}$$

Similarly, the negative affector can be given by:

$$A_{neg} = U_f - [(U_{\max}+1)-(U_f+1)]\frac{(C/K^-)^{m^-}}{1+(C/K^-)^{m^-}}. \quad \text{(A15)}$$

Applying the function composition operation, $f = A_{pos} \circ A_{neg}$, yields:

$$f(C) = (U_i+1)+\left[(U_f+1)-[(U_{\max}+1)-(U_f+1)]\frac{(C/K^-)^{m^-}}{1+(C/K^-)^{m^-}}-(U_i+1)\right]\frac{(C/K^-)^{m^-}}{1+(C/K^-)^{m^-}}. \quad \text{(A16)}$$

After some manipulation of this equation, we find that Eq. (A16) is identical to Eq. (A1), if the parameters of Eq. (A1) are subjected to the following set of transformations:

$U_i \mapsto U_i + 1$, (A17a)

$U_f \mapsto U_f + 1$, and (A17b)

$U_{\max} \mapsto U_{\max} + 1$. (A17c)

The result of Eqns. (A17a-c) is intuitive, given that we have merely shifted the y-axis of the biphasic curve, which does not otherwise affect its shape. Moreover, this shifting

does not change any of the concentration-concentration correlation functions identified by comparing the biphasic equation to a (novel) sigmoid equation (see the main text), given the sigmoid has also been equally shifted, which ensures that chemical-dependent concentration-response functions remain on an equal footing for comparison.

Appendix B:

Response Function Normalization Methods

Normalization of the sigmoid and biphasic response functions

Some response functions, such as a population's mortality, may be experimentally measured such that the control population suffers losses from expected effects, such as the normal aging process, or through additional adverse effects, such as disease. For such cases, a fitted response curve, such as mortality, $f(C)$, may not provide the intuitive expectation $f(C=0)=0$. (For an example of this effect, refer to the experimental data of Fig. 4 in the main text.) Additionally, a chemical compound experienced in large dosages or exposures may not be strictly fatal: $f(C=\infty)<1$. These effects may also depend on the time-scale of the measurements. Even so, it may be advantageous to normalize the fitted concentration-response curves to a fixed interval, from which the concentration-concentration correlation function can be reliably identified. Thus, we seek a set of equations that modify the values of the empirically fitted parameter values of both sigmoid and biphasic curves, such that the relative shape of the concentration-response function is preserved under a dilation or contraction of the response-axis.

Scaling relationships for parameters of the sigmoid curve

An example of a sigmoid curve is given by Fig. C(a). Our goal here is to determine new values for the parameters V_i and V_f, which we respectively label by $V_i' = \gamma$ and $V_f' = \alpha$

, that guarantee the response axis (i.e. y-axis) of the sigmoid curve is bounded on the interval $[\alpha, \gamma]$, which is fixed. To check whether simply assigning these values is sufficient or not, we are compelled to obtain an equation that maps values from one response-axis to the normalized one. To achieve this we note, that from Fig. C, the following equation holds:

$$\frac{x' - \gamma}{\alpha - \gamma} = \frac{x - V_i}{V_f - V_i}; \tag{B1}$$

Solving this equation for $x'(x)$ gives the transformation for any point along the y-axis:

$$x'(x) = \underbrace{\frac{\alpha - \gamma}{V_f - V_i}}_{\text{slope}} x + \underbrace{\gamma - V_i \frac{\alpha - \gamma}{V_f - V_i}}_{\text{y-intercept}}. \tag{B2}$$

Evaluating this equation at the endpoints confirms the desired endpoints: $V_i' = \gamma$ and $V_f' = \alpha$.

Scaling relationships for parameters of the biphasic curve

A similar methodology which led to Eq. B2 can be used to find scaled values for the initial and final levels of the sigmoid and biphasic concentration-response functions. As above, normalizing both curves in this way restricts descriptions to an effective response, rather than to absolute levels, which is a common practice in when the response levels are arbitrary or not standardized. In such situations a common concentration measure is the EC_{50}, which is the concentration that corresponds to a

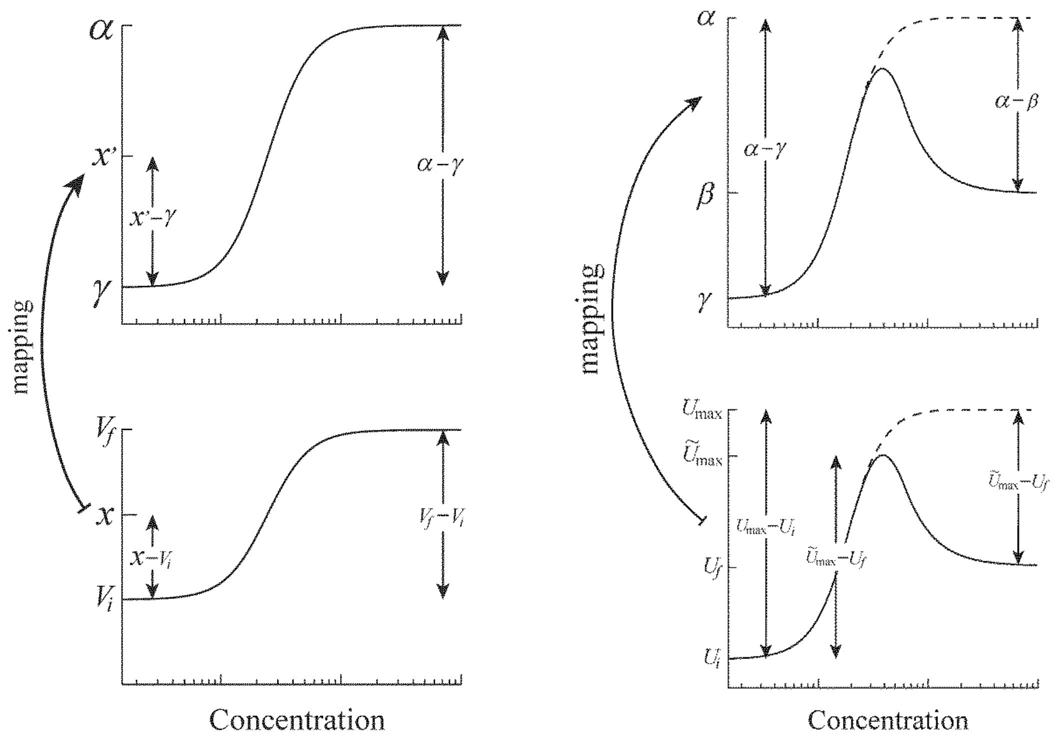

(a) **(b)**

Figure C. Normalization of the (a) sigmoid or (b) biphasic concentration-response functions. The bottom panel represents the non-normalized curve, while the top panel illustrates the result after normalization.

median (for a population), or half-maximal state level for the (potentially) nonlethal effect.

While it is straight-forward to normalize the sigmoid equation (see previous section), it is not clear which value along the concentration-response curve should be normalized against the maximal value of the sigmoid response. There appear to be two choices: (1) the maximum/minimum of the concentration-response intermediate to the initial and final levels; or (2) the final level of the concentration-response. Here, we proceed by adopting the viewpoint of item (1).

Figure C(b) illustrates this methodology. The way to derive the normalization transformations are similar to the method of the previous section, except that with the biphasic function there are three choices: (i) $U_i > U_f$; (ii) $U_i < U_f$; (iii) $U_i = U_f = U$. For the first restriction, (i), we have:

$$U'_f = \gamma \text{ ,} \tag{B3a}$$

$$U'_i = \frac{\gamma\left(\tilde{U}_{\max} - U_{\max}\right) + \alpha\left(U_{\max} - U_f\right)}{\tilde{U}_{\max} - U_f} \text{ , and} \tag{B3b}$$

$$U'_{\max} = \frac{\gamma\left(\tilde{U}_{\max} - U_i\right) + \alpha\left(U_i - U_f\right)}{\tilde{U}_{\max} - U_f} \text{ .} \tag{B3c}$$

For the second restriction, a similar result can be found:

$$U'_i = \beta \text{ ,} \tag{B4a}$$

$$U'_f = \frac{\beta\left(\tilde{U}_{\max} - U_f\right) + \alpha\left(U_f - U_i\right)}{\tilde{U}_{\max} - U_i} \text{ , and} \tag{B4b}$$

$$U'_{\max} = \frac{\beta\left(\tilde{U}_{\max} - U_{\max}\right) + \alpha\left(U_{\max} - U_i\right)}{\tilde{U}_{\max} - U_i} \text{ .} \tag{B4c}$$

Finally, the transformation equations valid for the last restriction, (iii), are:

$$U'_i = \gamma \text{ ,} \tag{B5a}$$

$$U'_f = \gamma \text{ , and} \tag{B5b}$$

$$U'_{\max} = \frac{\gamma\left(\tilde{U}_{\max} - U_{\max}\right) + \alpha\left(U_{\max} - U\right)}{\tilde{U}_{\max} - U} \text{ .} \tag{B5c}$$

Figure D Illustrates the overall result of the application of Eqns (B3)-(B5).

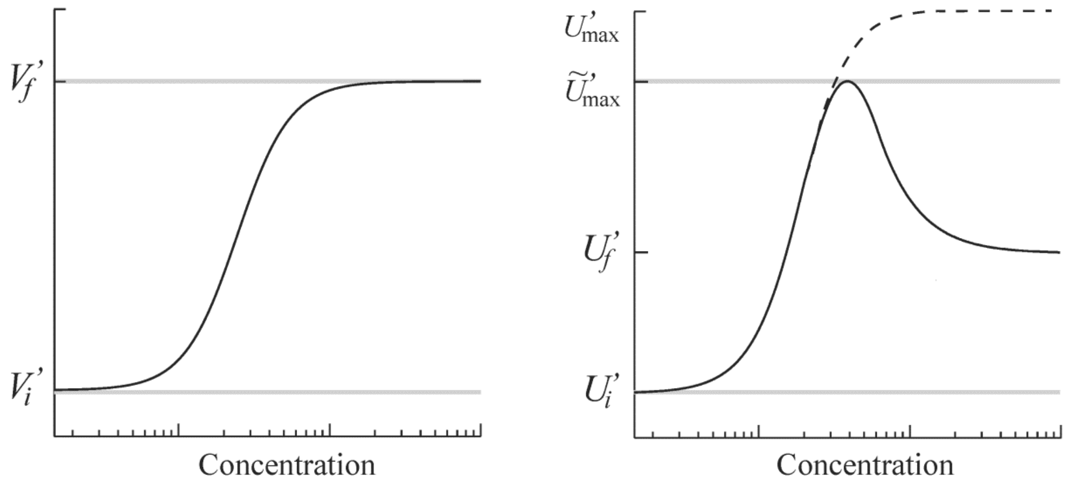

Figure D. Result of the normalization between sigmoid concentration-response curve (left panel) and a biphasic concentration-response function (right panel), using equations (20-22).

Table A. Parameter values for the biphasic curve illustrated (and modeled) in Fig. B.

Symbol	Unit	Value
U_i	Unit Response	1
U_f	Unit Response	0.1
$U_{\max}$	Unit Response	10
K^-	Unit Concentration	1
K^+	Unit Concentration	10
m^-	Unitless	2
m^+	Unitless	1
